# X International Symposium. 20 years advances in Oral Cancer, Bilbao, Spain. 3-5 July 2024

**DOI:** 10.4317/medoral.1122335667801

**Published:** 2024-07-15

X International Symposium. 20 years advances in Oral Cancer, 3-5 Julio 2024, Abstracts of the scientific communications follow.

