# Supplementary material for: X International Symposium. 20 years advances in Oral Cancer, Bilbao, Spain.: 3-5 July 2024
Source: Med Oral Patol Oral Cir Bucal. 2024 Jul 15;29(Suppl 1):S1–S80. doi: 10.4317/medoral.1122335667801 (PMC11354817; doi:10.4317/medoral.1122335667801)
Supplement: Related texts

# Abstract: Gingival spindle cell carcinoma with rapid growth in a middle aged man. Case report

**Authors:** C. Vera Abara, C. Torres Buscaglione, J. González Mardonez

**Affiliations:** *Herminda Martín Clinical Hospital, Chillán, Chile. Email: covera@ug.uchile.cl*

## Objectives

 Spindle cell carcinoma (SCC) is a malignant neoplasm, classified as a variety of the squamous cell carcinoma. Rare, it usually affects men in the upper aerodigestive tract and it has been associated with tobacco and alcohol. Histopathologically has normally a biphasic pattern, with a sarcomatous and a carcinomatous component. We aim to present a rare and challenging case of a spindle cell tumor in the oral mucosa.

## Case Report

 A 53 y-o-man with no comorbidities, heavy smoker, presents with a 5-months-evolution exophytic mass, with granular and ulcerated surface, arising from the right maxillary gingiva and palate mucosa, at least 2.5 cm length and slightly symptomatic. An incisional biopsy was taken.

## Results

 The biopsy showed a spindle cell proliferation, with diffuse positivity for KAE 1/3, MYOD1 and Vimentin as other rabdomioblastic differentiation markers. The diagnosis was a SCC, with heterologous differentiation. The CT scan showed a slight loss of cortical bone density and no suspicious lymph nodes. The patient was referred to the Maxillofacial Unit for resection.

## Conclusions

 SCC shows an aggressive behavior, tends to recur and its histopathology can be challenging, hence resorting to immunohistochemical techniques is necessary for distinguishing it from other spindle cell entities.

# Abstract: Mandibular chondroblastic osteosarcoma arising in a former site of excised central giant cell garanuloma; A rare case report in a woman

**Authors:** D. Alfatafta, N. Dar-Odeh, O. Abu-Hammad

**Affiliations:** *Department of Oral and Maxillofacial Surgery, Oral Medicine, and Periodontics, School of Dentistry, The University of Jordan, Amman, Jordan. Email: dr.dawlat.ft@outlook.com*

## Introduction

 Osteosarcoma is the most common malignancy of mesenchymal cells mostly originating within long bones constituting 50-60% of all primary bone tumors. Chondroblastic osteosarcoma is a subvariant of osteosarcoma; a rarely detected malignant bone tumor in the jaws which constitutes only 6% of all osteosarcomas. Timely diagnosis and treatment of this tumor may be adversely affected by the low grade development process and the close clinical similarity with other reactive or inflammatory lesions.

## Case Report

 This report describes a case of chondroblastic osteosarcoma that developed in the mandible of a 55-year-old woman. The tumor presented as a slowly growing swelling in the anterior mandibular gingiva. Ten years previously a gingival mass that was histologically diagnosed as central giant cell granuloma affecting the same area was excised. Preoperative radiological assessment using CBCT and histological examination of incisional biopsy were performed. This case report describes the clinical features, diagnostic approach and treatment outcomes. It further discusses the challenges in early diagnosis of this rare tumor. These challenges include the past history of a benign lesion of giant cell granuloma in the same area and the fact that it appeared in the mouth of a female patient which is inconsistent with the associated male prevalence of this tumor.

# Abstract: Use of obturators in postmaxilectomy patients. Presentation of two clinical cases

**Authors:** F. Alegria Riquelme, H. Slimane, C.J. Omaña Cepeda, P. Castañeda Vega, C. Moreno Soriano, C.S. Montoya Muñante, E. Jané Salas

**Affiliations:** *Faculty of Medicine and Health Sciences. UFR Dentistry, Bellvitge University Campus. University of Barcelona. Spain. Email: fiofeli12@gmail.com*

## Introduction

 A palatal obturator is a prosthesis that incorporates an obturator part that penetrates the edges of the defect seeking its peripheral closure at the oral level. They are indicated in cases of communications in the maxillary region, caused by surgical resection of tumors of the hard and/or soft palate, trauma or congenital malformations in which surgical reconstruction could not be performed or has failed. Objectives: To demonstrate the efficacy of palatal obturators as a rehabilitative alternative in patients who have undergoing maxillectomy.

## Case Report

 72-year-old man and 75-year-old woman with a history of OSCC of the upper jaw treated with partial maxillectomy; In both cases there were 2 failed attempts to close the defect using skin grafts, presenting a permanent orosinus communication. For the review, PubMed was searched using the keywords "obturators" AND "maxillectomies" published during the last 10 years; Of a total of 204 articles from the initial search, 4 articles related to the topic were obtained.

## Conclusions

 The main objective when designing a palatal obturator is to avoid orosinus communication, preserve the remaining teeth and tissues and provide comfort, function and aesthetics. It is an adequate and simple alternative to temporarily or permanently solve oral communications.

# Abstract: Chronic mechanical irritation as an etiological factor of oral cancer. Clinical case

**Authors:** L. Álvarez Iglesias, M. Pérez-Sayans, P. Gándara Vila, M. Somoza Martín, A. García García, B. Blanco Carrión

**Affiliations:** *Máster de Medicina Oral, Cirugía e Implantología. Universidad de Santiago de Compostela. Email: fiofeli12@gmail.com*

## Introduction

 Oral squamous cell carcinoma (OSCC) is the most frequent malignant neoplasm in the oral cavity. Its multifactorial etiology includes tobacco, alcohol, HPV infection... A little-studied factor could be chronic mechanical irritation (CMI).

## Objectives

 To present a clinical case of an uncommon lesion.

## Case Report

 81-year-old female patient with upper and lower complete prosthesis with no medical history of interest. She presented a lesion in the left jugal mucosa, atrophic, firm, asymptomatic and of unknown evolution. As differential diagnoses: traumatic ulcer, oral lichen planus, malignant tumor. An incisional biopsy was taken, which gave OSCC.

## Results

 IMC is described as the repeated low intensity action by an oral agent, such as malpositioned, broken teeth or misaligned prostheses. The most common areas are dorsum of tongue and jugal mucosa. These lesions are usually asymptomatic (long evolution period) and present with erythema, atrophy, ulceration, keratosis, hyperplasia or fibrosis. This persistent inflammatory state is the one that can allow the absorption of chemical carcinogens or increase cell proliferation, promoting oral carcinogenesis.

## Conclusions

 there are not enough studies to confirm that CMI per se is capable of causing cancer but it could be a promoter and progress or in oral carcinogenesis.

# Abstract: Developmental enamel defects as a consequence of early cancer treatment in a pediatric patient. Clinical case

**Authors:** B. Cordeiro Amarante, K.M. Pinto e Carvalho Rezende, K. Vaz, B. Marotta, M. Bönecker

**Affiliations:** *Department of Orthodontics and Pediatric Dentistry, Dental School, University of São Paulo (USP), Brazil. Email: bruamarante95@gmail.com *

## Introduction

 The treatment of childhood cancer has brought significant advances in survival; however, it has been associated with various long-term complications, including defects in the development of dental enamel.

## Case Report

 This case evaluated the effects of oncological treatment on a pediatric patient, who initiated chemotherapy and radiotherapy at 10 months of age due to non-metastatic high-grade undifferentiated SNC Glial Tumor. Treatment began with the removal of the initial tumor in April, 2016. However, there was a recurrence, and the patient underwent further surgeries 6 months later in October. Due to the recurrence, the patient underwent chemotherapy in November, 2016 and June, 2017. Further recurrences were diagnosed, and the patient required 2 sessions of radiotherapy in December, 2017 and May, 2018. The treatment and follow-up were conducted at the Institute of Pediatric Cancer Treatment in São Paulo, Brazil. Enamel hypoplasia in upper central incisors emerged after permanent teeth eruption at 6 years, likely due to radiotherapy during tooth development. Prophylactic dental care involved regular cleanings and fluoride varnish. Enamel hypoplasia poses aesthetic concerns, requiring future restorative treatment.

## Conclusions

 Understanding these defects is crucial for post-treatment dental management, highlighting the need for interdisciplinary collaboration between pediatric oncologists and dentists to offer personalized care.

# Abstract: HIV-associated plasmablastic lymphoma: A Case report

**Authors:** F. Arisa Petitbò, A. Torrejon Moya, N.V. Vilaplana, A. Estrugo Devesa, J. López-López

**Affiliations:** *Faculty of Dentistry. University of Barcelona (UB). Master of Medicine, Surgery and Oral Implantology. Campus Bellvitge, Hospitalet de Llobregat, Barcelona. Email: fredericarisa@gmail.com*

## Objectives

 In such a fast-growing and aggressive pathology as HIV-associated plasmablastic lymphoma it is essential to make an early diagnosis to establish a rapid and effective treatment to improve the patient's prognosis and life expectancy.

## Case Report

 We present a 36-year-old male patient from Morocco, a smoker of 20 cigarettes a day and consumption of 2 SD with no known medical allergies and a history of gonococcal infection and condyloma acuminata on the penis glans. The patient came to the clinic to evaluate a lesion of 2 months of evolution, exophytic with an ulcerated surface that extended from teeth 2.3 to the maxillary tuberosity.

## Results

 After completing the anamnesis, an incisional biopsy was taken with the histopathological result of diffuse lymphoproliferation in a starry sky pattern; Ki67 close to 100% and increased p53 expression of 30%. Serologies concluded HIV and EBV positive. The hemogram shows a CD4 T-lymphocyte count of 168/mm3 and a viral load of 99,000 copies/ml.

## Conclusions

 The presentation of this clinical case highlights the fundamental role of dentists, stomatologists, and maxillofacial surgeons in the diagnosis of this pathology. Commitment and awareness are essential for optimal health practice.

# Abstract: Oral squamous cell carcinoma in the floor of the mouth: A Case report

**Authors:** M. Arguimbau Coll, D. Sánchez Cousiño, C. Jorva García de Casasola, C. Polis Yanes, J. López-López

**Affiliations:** *Faculty of Medicine and Health Sciences - Master of Oral Medicine, Surgery and Implantology - University of Barcelona. Email: m.arguimbaucoll@gmail.com*

## Objectives

 To decrease the diagnostic interval and reinforce cessation of toxic habits.

## Case Report

 60-year-old male attends the Master of Medicine, Surgery and Oral Implantology referred from undergraduate to evaluate a lesion. He has a history of hypertension, angina pectoris and type 2 diabetes mellitus. He is also a pack-a-day smoker and a former alcoholic. He presents a single tumor lesion, located in the midline of the floor of the mouth, 1cm in diameter, pink coloration, irregular surface with sessile implantation, indurated and asymptomatic. A presumptive diagnosis of OSCC was established and was referred to the maxillofacial surgery service of the University Hospital of Bellvitge for evaluation.

## Results

 A histopathological test and a CT scan of the neck with contrast is performed. The diagnosis was confirmed as "infiltrating squamous cell carcinoma grade 2", without lymph node infiltration (T1N0M0). It is decided to perform excision of the primary lesion with bilateral cervical lymph node emptying.

## Conclusions

 The prognosis of the patient depends among many factors on the tumor stage, so we should focus on primary prevention, motivating patients to stop smoking, and on secondary prevention, early diagnosis during routine examinations.

# Abstract: Oral Lichen Planus: about a case

**Authors:** J. Barrantes, M. Mesquida, B. Tejedor, A. Torrejón, V. Schiavo, J. López‑López

**Affiliations:** *Master of Medicine, Surgery and Implantology - University of Barcelona. Spain. jhonellybr21@gmail.com*

## Introduction

 Oral lichen planus is a chronic inflammatory disease of the mucosa. Although the rate of malignant transformation is low, (1-3%), early diagnosis and follow-up of such patients is crucial.

## Objective

 Present a clinical case of long-evolving lichen planus.

## Case Report

 A 51-year-old female who came t referred by her dentist for the control of lesions clinically and histopathologically compatible with oral lichen planus, without control since 2011. The patient reports discomfort when eating acidic, cold or hot foods. She is a patient who smokes about 6 cigarettes a day, with no known allergies. As a medical history of interest, she suffers from oral and vaginal lichen planus. We observed desquamative gingivitis, as well as multiple lesions. On the lingual dorsum there is a white plaque with a 7mm papule in the center; in the lingual belly a 5mm exophytic lesion. A white plaque with a reticular pattern compatible with Wickham's striae is evident in the left buccal mucosa. It was decided biopsie incisional in order to obtain a histopathological diagnosis.

## Conclusions

 Once the histopathological result was obtained, compatible with oral lichen planus. So, we decided to perform controls every 3-6 months, during the first year to observe possible changes in the lesion.

# Abstract: Surgical reconstruction of hard palate following resection of mucoepidermoid carcinoma: Case report

**Authors:** L.I. Silva Cardoso, S.E.S. Maia, J.V. Lemos Ventura, R. Sales de Sá, E. Martins Pereira, I. Araújo Oliveira Consolaro, A. Consolaro, T. Santana

**Affiliations:** *Universidade CEUMA, São Luís, Maranhão, Brasil. Email: laisinescardoso@gmail.com*

## Objectives

 describe the surgical reconstruction approach following a mucoepidermoid carcinoma resection in the hard palate.

## Case Report

 Female patient, thirty-six years old, diagnosed with mucoepidermoid carcinoma in the right anterior palate, approximately 40 days of evolution, lesion measuring 5mm in diameter, referred for surgery together with the head and neck surgeon. The resection was performed with a 1cm safety margin in the radius of the lesion, leaving a defect of approximately 3cm in diameter. The palate was immediately reconstructed with a buccinator muscle flap associated with a mucoperiosteal flap from the palate on the left side, applying the principles of palatoplasty. The patient remained under follow-up for ten months, underwent 25 radiotherapy sessions and made progress. Palate reconstruction after tumour resection is technically arduous surgery and its success depends on the size of the defect left after resection.

## Conclusions

 Therefore, it is possible to conclude that applying classical surgical techniques of palatoplasty and local flaps of the oral cavity is effective in closing post-resection defects of malignant tumours in the palate.

# Abstract: Recurrence of an oral squamous cell carcinoma of the tongue in the same location 17 years later

**Authors:** M.L. Castro Rodríguez, A. Blanco Carrión, E. Otero Rey, P. Gándara Vila, M. Pérez-Sayans, A. García García, M. Somoza Martín, T. García Carnicero

**Affiliations:** *Máster de Medicina Oral, Cirugía Oral e Implantología. Universidade de Santiago de Compostela. Email: marialuisa.castro.rodriguez@rai.usc.es*

## Case Report

 An 80-year-old female patient, who is a non-smoker, presents with a medical history of depression, arterial hypertension, and a well-differentiated squamous cell carcinoma (SCC) of the oral cavity diagnosed in 2007. Despite attending periodic check-ups every six months, a clinical examination in 2023 revealed an oral white plaque on the right lateral border of the tongue and a slightly indurated erythematous lesion in the same area with a one-month history, both asymptomatic. Based on these findings, an incisional biopsy of the right lateral border of the tongue was performed, resulting in a histopathological diagnosis of a well-differentiated SCC.

## Results

 Several articles in the literature have described recurrences of lesions in the same location. Most agree that this second primary/recurrent SCC typically occurs within the first 5-10 years following the onset of the primary tumor.

## Conclusions

 SCC is the most common neoplasm of the oral cavity. The anatomical site most frequently affected by this tumor is the tongue, emphasizing the importance of conducting regular follow-up examinations throughout the patient's life and monitoring potential risk factors.

# Abstract: Lingual ulcer as a presentation of OSCC on the tongue: a case report

**Authors:** P. Suarez Pérez, M. Costa Tort, A. Torrejón Moya, E. Jané Salas, J. López-López

**Affiliations:** *Facultad Medicina Ciencias de la Salud, Odontología, Universidad de Barcelona. Máster de Medicina Cirugía e Implantología Oral (MCIO). Barcelona. Email: suarez.perez.p@gmail.com*

## Objectives

 Highlight the importance of a correct and early diagnosis of oral ulcerations.

## Case Report

 A 38-year-old male patient, with no relevant medical history or toxic habits, was referred to evaluate an ulcer on the lateral edge of the tongue with no associated etiological factor. On clinical examination we observed an ulcer on the right lateral edge of the tongue measuring about 5x4mm, covered by a yellowish-white layer and surrounded by a leukoerythematous halo, round in shape, well delimited but with slight induration on palpation. Nodal palpation was within normal parameters. The patient reported mild discomfort with acidic foods. The evolution time was 2-3 months. Due to the suspicion of a possible neoplastic cause, and having eliminated all possible traumatic factors, it was decided to perform an incisional biopsy.

## Results

 In the pathological study, infiltration of the subepithelial connective tissue and muscle was observed due to epithelial neoplasia with architectural disorder and severe cytological atypia. The diagnosis was grade 3/3 infiltrating squamous cell carcinoma.

## Conclusions

 The presence of a malignant process should be suspected in any ulcer that persists for more than 15 days and such a case should be urgently referred for appropriate evaluation.

# Abstract: Oral leukoplakia: Approach to a potentially malignant lesion and management strategies for oral cancer prevention. A Case report

**Authors:** E.I. Ouaghmiri Nissrine, E. Carrasco Núñez, R. Cabezas Turrado, S. Egido Moreno, E. Jané Salas, J. López-López

**Affiliations:** *Faculty of Medicine and Health Sciences (Dentistry) - Master of Oral Medicine, Surgery and Implantology - University of Barcelona. Email: elouaghmirinissrine@gmail.com*

## Objectives

 To identify, control, and prevent the malignant transformation of oral leukoplakia through the management of risk factors and therapeutic strategies.

## Case Report

 A 65-year-old male with a history of smoking and alcoholism presented with a bilateral white plaque on the retro-commissural buccal mucosa with reddish areas, firm consistency, and a size of 2x3 cm, of 1-year evolution. Several differential diagnoses were considered, including leukoplakia, chronic hyperplastic candidiasis, and oral squamous cell carcinoma. After ruling out possible underlying causes such as smoking and administering antifungal treatment in case of suspected fungal infection without response, a biopsy was performed for a definitive diagnosis. The histopathological report revealed epithelial hyperplasia, hyperkeratosis, and parakeratosis, without criteria of dysplasia confirming the diagnosis of homogeneous leukoplakia. Follow-up appointments were scheduled.

## Conclusions

 Leukoplakia is a potentially malignant disorder with a 9.8% risk of malignant transformation. Early detection, appropriate therapeutic management, and access to education and dental services are essential to improving clinical outcomes and patient quality of life, highlighting the importance of prevention in the fight against oral cancer.

# Abstract: Risk of developing oral squamous cell carcinoma in former smokers: Clinical case

**Authors:** F. Elwy Khaled, J.R. Merino López, F. Arisa Petitbó, A. Torrejón Moya, E. Jané Salas, S. Egido Moreno, J. López-López

**Affiliations:** *Faculty of Medicine and Health Sciences (Dentistry). Medicine, Surgery and Oral implantology Master. University of Barcelona. Email: faroukelwy@gmail.com*

## Objectives

 To evaluate the clinical presentation, diagnosis, and histopathological characteristics of a tongue lesion in a former smoker, and to discuss the associated risk of squamous cell carcinoma in long-term former smokers.

## Case Report

 A 54-year-old male, former smoker of 5 cigarettes/day for 25 years, seeks endodontic treatment for tooth 1.7. Referred from endodontic service to University of Barcelona's Master of Oral Medicine, Surgery, and Implantology to assess a 2.5 cm x 1.2 cm lesion on the right lateral tongue border. Poorly defined borders, palpable induration, symptomatic, no associated lymphadenopathy, lesion unresponsive to scraping. With a presumptive diagnosis of erythro-leukoplakia. It is decided to polish edges coinciding with lesion and conduct excisional biopsy. The histopathological report revealed squamous cell carcinoma grade 2/3 with a maximum invasion depth of 1.5 mm, severe epithelial dysplasia adjacent to the lesion, and infiltration of the lesion affecting one of the resection margins of the specimen.

## Conclusions

 Although smokers have a higher risk of developing squamous cell carcinoma, the risk of developing squamous cell carcinoma in former smokers who smoked for more than 20 years is still higher than in never-smokers.

# Abstract: The importance of early treatment of "high risk" oral potentially malignant disorders (OPMD)

**Authors:** C. Ferdeghini, C. Digregorio, G. Beltramini, S. Fiori, A. Troiano, M. Vimercati, M. Roghi

**Affiliations:** *Istituto Stomatologico Italiano – Milano. Italia. Email: cferdeghini@virgilio.it*

## Objectives

 This poster shows two case report of high risk OPDM treated in early stages. It aims to explain the importance of early diagnosis and management of hish risk OPDM.

## Material and Methods

 We selected 2 patients with tongue speckled lesions. For patient 1, after clinical examination, with the aid of autofuorescence, we took a punch biopsy that revealed G2 OSCC. For patient 2, we took 2 biopsies that revealed OLP, with no evidence of dysplasia. 18 months (and several controls) later, a new biopsy showed a squamous cell carcinoma in situ.

## Results

 Our referring oncologist proceeded to remove the neoplastic lesions in both patients. In both case intra-op histology confirmed areas ranging from severe dysplasia to G1 OSCC. Adjuvant treatment was not deemed necessary and the 2 patients were included in a follow-up program.

## Conclusions

 OSCC is a pathology whose 5-year survival rate is less than 50%. Early diagnosis therefore allows us to identify the cancerous lesion in its initial stage and guarantees patients a less invasive therapy with a better prognosis.

# Abstract: Application of photobiomodulation in lymphedema secondary to head and neck cancer treatment

**Authors:** C. Giacco, Á. Castrillo, V. Flück, J. Gimenez

**Affiliations:** *Hospital Municipal Oncología María Curie, Buenos Aires, Argentina. Hospital de Odontología Dr. Ramón Carrillo, Buenos Aires, Argentina. Email: claugiacco@gmail.com*

## Objectives

 to present a clinical case of lymphedema secondary to treatment of head and neck cancer (HNSCC) and proposal of photobiomodulation protocol.

## Case Report

 74-year-old female patient with a history of epidermoid carcinoma of the floor of the mouth with 3 events during the years 2017, 2020, 2021 treated with surgery, lymphadenectomy and IMRT 70 Gy + Cisplatin 100 mg/m2. At 2 months she developed bilateral facial LSCCC. Photobiomodulation was planned: diode laser, Therapy EC DMC 660nm - 808nm - 100 mW - 10 sessions, fluence 18 joules/cm2, weekly appointment with photographic controls.

## Results

 Good clinical response to treatment with no recurrence of LSCCC was evidenced.

## Conclusions

 HNSCC according to the literature is little studied, however its prevalence is estimated between 50 and 75%. It can lead to infections, cellulitis and long-term aesthetic, functional and psychosocial complications that are often irreversible. Photobiomodulation is one of the reported treatments very well tolerated and with good prospects of resolving this complication as in the case presented. More multicenter clinical trials, essential to improve therapeutics and quality of life of patients, are needed.

# Abstract: Adenosquamous carcinoma in maxillary gingiva: Case report

**Authors:** L.F. López Hernández, J.S. Zepeda Nuño, J. Contreras Núñez, A.E. Sánchez Becerra

**Affiliations:** *Master's in Pathology and Oral Medicine, Department of Integral Dental Clinics, Health Sciences University Center, University of Guadalajara. Email: elizabeth.sanchez@academicos.udg.mx*

## Introduction

 Adenosquamous carcinoma is a rare high-grade malignant epithelial neoplasm that arises from the superficial epithelium. In the head and neck region, the larynx is the most frequently affected site, followed by the oral cavity, with the tongue and gums being the most affected areas.

## Case Report

 A 68-year-old male patient presented with an oval-shaped neoplasm approximately 3 cm in diameter, with a sessile base and smooth surface featuring telangiectasias, located in the anterior region of the upper alveolar process. The lesion had been evolving over four weeks with associated pain and rapid growth, secondary to the extraction of teeth 12 and 13. A periapical radiograph revealed a radiolucent area with diffuse borders. The patient reported unquantified weight loss, anorexia, asthenia, and adynamia over the past month. Upon questioning, he mentioned smoking 20 cigarettes a day for the past 20 years. An incisional biopsy was performed for histopathological examination. The histopathological findings were consistent with adenosquamous carcinoma. The origin of this neoplasm is still debated. The WHO’s 2022 classification of head and neck tumors defines it as a malignant neoplasm arising from the surface epithelium, showing both squamous and glandular differentiation. Little is known about the etiopathogenesis of this entity, primarily due to its rarity and lack of studies. However, smoking and alcohol consumption are believed to play significant roles in its pathogenesis.

## Conclusions

 This malignant entity poses a diagnostic challenge due to the need to identify various histopathological criteria, especially in incisional biopsies, and its histopathological similarities with other malignant neoplasms.

# Abstract: Field cancerization. A case report

**Authors:** M.J. Lucero Berdugo, F. Molina Miñano, A. Ferrández Pujante, C. Castillo Felipe, R. Bello Sánchez, J.F. Martínez Lage-Azorín, L. Párraga Linares, M. Cobos Martín, M.F. García Luna

**Affiliations:** *Facultad de Ciencias de la Salud. Universidad Catoìlica San Antonio de Murcia (UCAM). Guadalupe. Murcia. Email: fmolina@ucam.edu*

## Case Report

 75-year-old male with no history of ADR, history of hypertension, no MD, morbid obesity, moderate alcoholism, former cigar smoker and lower PPR. In 2017 he underwent surgery at the MFS of the San Juan de Alicante clinical hospital for squamous cell carcinoma on the dorsum of the tongue and left CSE, removing the lesion with margins and direct closure. In the same operation, an excisional biopsy of a 2 cm lesion on the right dorsum of the tongue was performed. The left lesion was diagnosed as well-moderately differentiated epidermoid carcinoma, staged T2N0, while the right lesion was diagnosed as squamous cell carcinoma. In the histopathological study, a homogeneous leukoplastic lesion was observed on the left floor of the mouth in the tongue of the mouth accompanying the surgical specimen. He was treated with adjuvant radiotherapy after assessment by the head and neck oncology committee. He underwent a second surgery to remove the lesion on the right dorsum of the tongue that same year. In 2019 the patient underwent surgery for squamous cell carcinoma of the throat at the IVO in Valencia with adjuvant QT and RT. The patient is currently free of lesions although with functional and aesthetic sequelae.

# Abstract: Locoregional progression of oral squamous cell carcinoma: when palliative treatment becomes the only option

**Authors:** L. Maftei Rusu, C. Omaña Cepeda, V. Schiavo Di Flaviano, A. Torrejón Moya, E. Jané Salas, J. López-López

**Affiliations:** *Faculty of Medicine and Health Sciences (Dentistry). University of Barcelona (UB). Master of Oral Medicine, Surgery and Implantology (MCIO). University of Barcelona Dental Hospital. Barcelona. Email: lorenac.maftei@gmail.com*

## Objectives

 To present a clinical case of locoregional recurrence of an oral squamous cell carcinoma (OSCC).

## Case Report

 A 78-year-old woman with a 9-year history of oral lichen planus, without toxic habits, was diagnosed with squamous cell carcinoma of the upper left maxilla with sinus involvement. A surgical excision of the lesion was performed and, two months later, the disease progressed locoregionally (T4N0), extending from the pterygoid process to the mandibular condyle and left temporomandibular joint, involving the lateral pterygoid muscle, affecting the middle cranial fossa, sphenoid wing, and the laterobasal wall of the orbit.

## Results

 OSCC represents up to 90% of malignant neoplasms arising in the oral cavity. In some cases, it may be preceded by potentially malignant oral lesions such as oral lichen planus. Locoregional progression is the most common presentation, with the percentage of metastasis to other organs being less than 15%. For N0-N1 tumors, the percentage of distant metastasis is less than 10%, with the main target organs being the lungs and bones.

## Conclusions

 Surgical treatment and complete cure of advanced-stage squamous cell carcinoma have a reduced prognosis. Sometimes, palliative treatment becomes the only option.

# Abstract: Clear cell odontogenic carcinoma of the mandible: report of two cases

**Authors:** R. Izzetti, F. Minuti, P. De Francesco, M. Nisi

**Affiliations:** *University of Pisa, Department of Surgical, Medical and Molecular pathology and Critical Care Medicine, Pisa, Italy. Email: filippominuti98@gmail.com*

## Objectives

 Clear cell odontogenic carcinoma (CCOC) is a rare malignant odontogenic tumor, with only 107 cases reported in the literature. Due to its infrequency, evidence on diagnostic criteria, treatment protocols, and prognosis of CCOC is scarce. This study describes two cases of mandibular CCOC treated with radical surgical approach.

## Case Reports

: A female patient aged 64 years was referred for the evaluation of an ulcerated gingival mass localized in the anterior right mandible. A 70-year old female patient had an osteolytic lesion with bicortical bone destruction on CT examination in the absence of clinical signs. Incisional biopsy was performed in both patients, and histopathology revealed a diagnosis of CCOC. Head and neck MR and total body PET-CT were performed to further investigate the lesion.

## Results

 Radical surgery was performed in both patients and involved hemimandibulectomy to achieve clear resection margins and lymph node dissection. Reconstruction of the postsurgical composite-tissue defect of the mandible was performed with a fibula osteoseptocutaneous flap. At follow-up (60 months for the first patient, 36 months for the second patient), no signs of recurrence were noted.

## Conclusions

 Radical surgical approach involving wide local excision combined with regional lymph node dissection appears effective in treating CCOC.

# Abstract: Lesion on dorsum of the tongue

**Authors:** F. Molina Miñano, M.J. Lucero Berdugo, A. Ferrández Pujante, C. Castillo Felipe, R. Bello Sánchez, J.F. Martínez Lage-Azorín, L. Párraga Linares, M. Cobos Martín, M.F. García Luna

**Affiliations:** *Facultad de Ciencias de la Salud. Universidad Catoìlica San Antonio de Murcia (UCAM). Guadalupe (Murcia). Email: fmolina@ucam.edu*

## Case Report

 68-year-old smoker female (30/day) and maxillary edentulous same to take a rehabilitation treatment with dental implants. She was diagnosed by her general practitioner as median rhomboid candidiasis and treated with antifungals (Nystatin and Fluconazole) for the last month. The lesion worsened to an ulcerated appearance in the midline of the lingual dorsum with raised edges, with painful and indurated areas in the peripheral site. An incisional biopsy was performed and the histopathological result presented by the oral and maxillofacial pathology diagnostic service (SDPOMF) diagnosed epithelial hyperplasia with hyperkeratosis, mucositis and chronic submucositis, fungal infection and moderate epithelial dysplasia. After one month, she was called for a follow up and the lesion was found to have healed.

# Abstract: Mantle cell lymphoma: clinical case

**Authors:** M.J. Mora Barba, P. López Jornet, F.J. Gómez García, J.A. Ruiz Roca, F. Galera Molero, J.F. Ros Madrid

**Affiliations:** *Medicina Oral. Universidad de Murcia. Murcia. España.Email: mjmorabarba@gmail.com*

## Introduction

 Mantle cell lymphoma (MCL) is a rare type of aggressive B-cell non-Hodgkin lymphoma that most commonly affects male adults, with an average age of 65 years. Patients with MCL may present with bone marrow involvement, lymph nodes, spleen and digestive system, with cytopenias.

## Objectives

 to present a clinical case of oral ulcerations in a patient treated with immunotherapy.

## Case Report

 63-year-old male, diagnosed with mantle cell lymphoma. P.A: allergic to anesthetics, bicuspid aortic valve; Gilbert syndrome; autologous bone marrow transplant. Relapse with Thrombopenia + constitutional syndrome. Referred by Hematology to the Oral Medicine Unit due to very painful major aphthae in the throat and tongue. After the examination, an incisional biopsy of the lingual ulcer (nonspecific ulceration) was performed and topical corticosteroid treatment was subsequently initiated with grinding of the edges of the molars without improvement. After consulting with Hematology, systemic treatment with deflazacort was instituted for neutropenic ulcers, showing notable improvement.

## Conclusions

 immunotherapy for treatment of MCL can produce cytopenias with secondary presentation of oral ulcerations. It has been observed that treatment with systemic corticosteroids produces a notable improvement in oral ulcers compared to topical corticosteroid treatment.

# Abstract: Minor salivary gland secretory carcinoma in a child: a rare case report involving the upper lip

**Authors:** B. Munhoz Marotta, G. Banacu de Melo, N. Souza de Andrade, D. Heguedusch, D. dos Santos Pinto Júnior, C. de Barros Gallo

**Affiliations:** *Stomatology Department of the School of Dentistry of the Universidade de Sao Paulo, Sao Paulo/SP – Brazil. Email: bmmarotta@outlook.com*

## Introduction

 Approximately 25% of all salivary gland tumors are diagnosed in minor salivary glands, mostly benign lesions at the palate of adult men.

## Case Report

 This report comprises a 9-year-old girl with a 1-year mass perceived after a trauma episode at her upper lip. Intraoral examination showed an asymptomatic and well-defined 2-cm fibrous submucosal nodule covered by normal oral mucosa. The excisional biopsy was performed with the hypothesis of pleomorphic adenoma and sialadenitis. The histological analyses revealed salivary gland neoplasm, composed of clear cells, sometimes with evident secretion and formation of cell sheets, tested for CK7, CK8, S100 and mammaglobin at the immunohistochemical study. The final diagnosis was secretory carcinoma, and the patient was referred for the oncological treatment that consisted in a complementary surgery without signs of residual lesion, and the patient remains without evidence of recurrence or metastasis in a follow-up period of 3 years. The secretory carcinoma is a rare tumor in minor salivary glands and in pediatric populations. And despite the low-grade malignancy behavior the study, this diagnose can be considered when dealing with circumscribed nodular lesions of minor salivary glands due to the potential worse prognosis related to delay or misdiagnosis.

# Abstract: Multifocal oral tumor lesion with a complex diagnosis in an HIV (+) patient

**Authors:** P. Antía Pose, J.M. Suárez Peñaranda, P. Boñar Álvarez, J.M. Aguirre Urizar, A. Blanco Carrión, P. Gándara Vila

**Affiliations:** *Master of Oral Medicine, Oral Surgery and Implantology. Santiago de Compostela University. Pathology and Oral Medicine, Department of Stomatology. University of the Basque Country/EHU. Email: aaantiapose@gmail.com*

## Objectives

 To present a complex tumor clinical case in an HIV (+) patient.

## Case Report

 54-year-old man, smoker, HIV (+), with a history of Kaposi's sarcoma and recurrent papillomas. In 2022 he presented a 2 cm sessile, erythematous tumor lesion in the vestibule and right jugal mucosa diagnosed as condyloma, and removed with a CO2 laser. In 2023, multifocal tumor lesions appear in the upper alveolar ridge, which are diagnosed as Papilloma and removed. In 2024, an exophytic tumor lesion appeared in the first quadrant gum, which was diagnosed as basaloid carcinoma and treated.

## Conclusions

 Basaloid carcinoma is a rare variant of oral squamous cell carcinoma, which has been related to HPV. This clinical case draws attention due to its relationship with HIV infection, its multifocality, location and biological behavior.

# Abstract: To repeat or not to repeat a biopsy? A clinical case report

**Authors:** R. Prat Riera, R. Ezzeddine Doughan, R. Cabezas Turrado, S. Egido Moreno, J. López-López

**Affiliations:** *Faculty of Medicine and Health Sciences [Dentistry] - Master of Oral Medicine, Surgery and Implantology - University of Barcelona. Email: remeiprat6@gmail.com*

## Objectives

 To help prevent malignancy of the leukoplakia.

## Case Report

 A 49-years-old male patient with a medical history of depression, medicated with mirtazapine and citalopram, allergic to penicillin, smoker of 20 cigarettes a day, came to the Dental Hospital of the University of Barcelona. He presented a single, raised lesion of multiple heterogeneous yellowish-white papules on the alveolar ridge of the first quadrant; 1cm in diameter, irregular borders, with poorly defined limits that did not detach on scraping and with slight discomfort on palpation. In September 2023, an incisional biopsy was performed and the result was papillomatous hyperplasia without atypia. In January 2024, the lesion persists and it is decided to perform a new biopsy, this time excisional, to rule out histopathological changes.

## Results

 The result is papillomatous hyperplasia with areas of severe epithelial dysplasia. When presenting margins with dysplasia, a new biopsy is performed, this time with trephine to obtain bone tissue and again results without dysplasia are obtained.

## Conclusions

 The role of the dentist is fundamental in detecting clinicopathological changes, triggering early detection of oral cancer, as late diagnosis of oral squamous cell carcinoma prevents successful treatment and favorable outcomes.

# Abstract: Proliferative verrucous leukoplakia, case report with 10 years of follow-up

**Authors:** V. Robles Oneto, A. Torrejon Moya, P. Roca Obis, E. Jané Salas, S. Egido Moreno, J. López-López

**Affiliations:** *University of Barcelona Dental Hospital. University of Barcelona. Email: vroblesoneto@gmail.com*

## Objectives

 To highlight the importance of monitoring patients diagnosed with Proliferative verrucous leukoplakia (PVL).

## Case Report

 A 48-year-old female patient, smoker of 2 cigarettes a day, was diagnosed with PVL in 2014. She has presented great variability of lesions during the last 10 years, according to the area of the affected oral mucosa, location of the lesions, and macroscopic appearance. This is why she has undergone several biopsies of the highest-risk areas, to anticipate possible malignancy.

## Results

 The patient underwent multiple biopsies during the 10 years of follow-up, obtaining results that led us to the same diagnosis of PVL.

## Conclusions

 Because of the aggressive behavior and its high probability of malignant transformation, early diagnosis and constant surveillance with periodic biopsies, are fundamental in the management of patients with PVL, especially when the lesions are constantly changing.

# Abstract: *In situ* melanoma of the oral mucosa. Clinical case

**Authors:** J.F. Ros Madrid, P. López Jornet, F.J. Gómez García, J.A. Ruiz Roca, F. Galera Molero, M.J. Mora Barba

**Affiliations:** *Medicina Oral. Universidad de Murcia. Email: josefidel.rosm@um.es*

## Introduction

 Oral mucosal melanoma is a rare malignant neoplasm that arises from the uncontrolled growth of melanocytes located in the basal stratum of the oral mucosa epithelium. Its incidence is 0.073% of all cancers. Approximately 80% of oral melanomas develop in the jaw, especially in the palate and alveolar gingiva. Lesions can range from typically pigmented, nonpigmented, single, or multiple.

## Objectives

 to present a clinical case about an in situ melanoma located in the oral mucosa.

## Case Report

 a 72-year-old woman with hypertension, without toxic habits, came to the consultation for a suspicious melanic pigmentation located in the vestibular attached gingiva in the premaxilla, of 5 years' duration. After clinical examination and complementary tests, an incisional biopsy was performed, with a diagnosis of “lentiginous "in situ" melanoma of the oral mucosa. After knowing the result, the patient was referred to the maxillofacial surgery service of her reference hospital.

## Conclusions

 oral mucosal melanoma is a rare neoplasm with an unfavorable prognosis, so early diagnosis and treatment is crucial. Any pigmented lesion in the oral cavity should be carefully examined and biopsied.

# Abstract: Hypocellular inflammatory myofibroblastic tumor: Case report

**Authors:** A.T. Avila Hernández, L. Hernández Belmont, A. Galicia Partida, E. Santos Jaimes

**Affiliations:** *Instituto Politécnico Nacional, México. Email: aviher23@gmail.com.*

## Introduction

 Our aim is to share our experience with an unusual case of Inflammatory Myofibroblastic Tumor (IMT), a rare neoplasm predominantly benign but occasionally it can exhibit aggressive and malignant behavior. It has been documented across a wide anatomical distribution, with 15% of cases reported in the head and neck region. Sinonasal and oral cavity IMTs tend to be clinically aggressive, with higher rates of recurrence and metastasis. Its varied histopathological characteristics pose diagnostic challenges.

## Case Report

 A 48-year-old female presented with a sessile, exophytic nodular lesion, ulcerated, 21 mm diameter in the left palatal region near to the alveolar mucosa of premolars. Radiological examination showed no evidence of bone involvement. An excisional biopsy was performed; the histological and immunohistochemical evaluation revealed a Hypocellular Inflammatory Myofibroblastic Tumor, with myxoid appearance and desmoplastic areas, exhibits classic myofibroblastic cells and reactive plasma cells, along with bone neoformation apparently reactive, a feature that to our knowledge has not been described in the histological patterns of this neoplasia. Strict follow-up was recommended.

## Conclusions

 IMT is a neoplasm with intermediate biological potential, characterized by a low risk of recurrence and metastasis. Our findings provide information that adds to the description of its histological patterns.

# Abstract: Large oral squamous cell carcinoma in the tongue in a young man, without associated risk factors

**Authors:** C. Torres Buscaglione, C. Vera Abara, C. Robles Aravena, R. Cabrera Rojas, A. Del Río-Salas, K. Devia Cutiño, C. Jarpa Antinao, C. Tagle Alegría

**Affiliations:** *Oral and MaxillofacIal Pathology, Padre Las Casas Hospital, Padre Las Casas, Chile. Email: ctb.patologiaoral@gmail.com*

## Introduction

 Oral squamous cell carcinoma (OSCC) accounts for less than 2% of cancer and more than 90% of oral malignancies. Associated risk factors are tobacco, alcohol, areca nuts, immunosuppression, human papilomavirus and advanced age. Commonly in over 60-y-o-men, it often arises as a painless, non-healing ulcer that can affect any part of the oral mucosa. The objective is to present a case of OSCC in the tongue, in a patient without associated risk factors.

## Case Report

 A 45-year-old man, non-smoker, non-alcohol consumer and with no history of morbidity, went to the Padre Las Casas Hospital with painful, ulcerated tumor on the left side of the tongue, with raised and indurated borders, measuring at least 3 cm and with 2 months of evolution. An incisional biopsy was taken.

## Results and Conclusions

 The diagnosis was confirmed by histopathology report, which showed a moderately differentiated OSCC, negative for p16 immunostaining. Referral to the Head and Neck Surgery Unit was made for resection. OSCC strongly impacts the patient's functionality, aesthetics and emotional state. The rapid evolution, intense symptoms, along with the lack of associated risk factors in this patient, makes us reflect on the parameters that we are used to seeing in the OSCC.

# Abstract: Possible factors related to dorsal tongue carcinoma

**Authors:** D. Yagudaev, A. Hefetz, A. Novikova, F. Yoram, C. Rakefet

**Affiliations:** *Department of Oral Medicine, Sedation and Imaging, Hadassah Medical Center, Hebrew University of Jerusalem, Israel. Email: dr.danielyagu@gmail.com*

## Introduction

 Dorsal tongue has a low incidence of Squamous cell carcinoma (SCC). We present a case with dorsal tongue carcinoma arising in a long-standing site of non-erosive lichen planus (LP).

## Case Report

 73-year-old patient, complained at one of his routine follow ups of a new symptomatic nonhealing ulcer on the dorsal tongue for a month. His medical background included Epilepsy and smoking for 25 years. In 2012 a buccal mucosa biopsy result was lichenoid reaction with mild dysplasia. During the 12 years of 24 follow-ups, reticular and sometimes erosive forms of LP were noticed at his dorsal tongue and buccal mucosa. At the recent examination, 4 mm symptomatic ulcer was noticed. Due to no response to 4 weeks of topical triamcinolone treatment biopsy was made, suspected and diagnosed with SCC. Later his son was also diagnosed by a dorsal tongue biopsy of LP.

## Conclusions

 High-risk patients (with OLP and smoking) should be under a lifelong follow up due to possible malignant transformation. The location of the SCC in the dorsal tongue is rare, but should be suspected when a high-risk lesion is identified. Familial oral LP was recently reported as suspected to be a risk factor to SCC.

# Abstract: Positive p16 in a patient with Proliferative Verrucous Leukoplakia associated with cellular senescence and not HPV infection

**Authors:** E.A. Georgakopoulou, D. Dimitris Veroutis, C. Kittas, V.G. Gorgoulis

**Affiliations:** *Molecular Carcinogenesis Group, Department of Histology and Embryology, Medical School, National and Kapodistrian University of Athens, Athens, Greece. Email: stomagiatros@gmail.com*

## Introduction

 HPV infection has a questionable role in the pathogenesis of PVL. This was a patient with multiple recurrences of PVL.

## Case Report

 this is a case of a female patient with Proliferative Verrucous Leukoplakia (PVL) which tested positive for p16 on immunohistochemistry , negative for HPV infection on PCR and positive for the senescence biomarker SenTraGor. To test in this case the relevance of possible HPV infection to the development of PVL we performed p16 IHC and PCR for HPV DNA.

## Results

 The p16 ICH tested positive but the PCR for HPV DNA did not confirm the presence of the virus. Subsequently we applied the SenTraGor senescence biomarker, which tested positive. This result may indicate that positive p16 may be related to cellular senescence and not to HPV infection.

# Abstract: Ultrastructural features of epithelial-mesenchymal transition (EMT) in early oral squamous cell carcinoma (OSCC)

**Authors:** C. Berga, P. Iruzubieta, T. Usón, M.A. Trigo, M.C. Junquera

**Affiliations:** *Faculty of Medicine, University of Zaragoza and Instituto Aragonés de Investigación Sanitaria. Instituto de Investigación Sanitaria Biogipuzkoa. Email: clinicadentalbergahidalgo@hotmail.es*

## Objectives

 Epithelial-Mesenchymal Transition occurs in certain types of carcinomas as a precursor stage to metastasis when epithelial cells receive different signals that lead to their detachment from adjacent cells, initiating metastasis after breaking the basal lamina. Based on our positive immunohistochemical findings for certain EMT markers, an ultrastructural study was carried out focusing on the presence of primary cilia and the disassembly of cell junctions in oral cancer keratinocytes.

## Material and Methods

 Five early-stage OSCC tissue specimens were used for the study. All samples were conventionally processed and analyzed using a JEOL JEM 1010 transmission electron microscope (80 Kw) and a GATAM-BIOESCAN camera to examine serial ultrathin sections.

## Results

 In early oral tumorigenesis, many tumour cells in mitosis reach the spinous layer. Some basal layer cancer cells show primary cilia that receive EMT signals, leading to widened intercellular spaces, small keratinocyte extensions, and loosening desmosomes. This result in interrupted cell junctions, loss of morpho-functional polarity and cancer cells adopting an elongated mesenchymal phenotype, allowing them to penetrate the basal lamina and spread to vessels.

## Conclusions

 Ultrastructural studies provide early insight into some of the processes that occur in early stages of tumorigenesis in oral cancer cells: primary cilia emission, loss of polarity and desmosomes disassembly.

# Abstract: Imaging markers of leukoplakia with Ultra-High Frequency Ultrasound

**Authors:** R. Izzetti, P. de Francesco, F. Minuti, M. Nisi

**Affiliations:** *University of Pisa, Department of Surgical, Medical and Molecular pathology and Critical Care Medicine, Pisa, Italy. Email: pierpaolo.defrancesco96@gmail.com*

## Objectives

 The aim of this study was to assess the ultrasonographic features of oral leukoplakia to identify imagine patterns associated with this disorder.

## Material and Methods

 Consecutive patients with a diagnosis of leukoplakia were enrolled and underwent intraoral ultra-high frequency ultrasonography examination (UHFUS), involving the application of 70 MHz ultrasound frequencies. Lesion thickness and mean echogenicity were evaluated. Color Doppler acquisi/on was performed by se7ng flow velocity at 1.98 cm/s in order to detect slower flow vessels. Vascular parameters included Peak Systolic Velocity (PSV) and Resistive Index (RI).

## Results

 Twenty patients (13 males, 7 females, mean age 61.5 years, SD 17.4) were enrolled. Epithelial thickness appeared increased in correspondence with leukoplasic lesions, and appeared as a hypoechoic homogeneous band with well-defined margins and separated from the underlying submucosa. The lesions presented a mean thickness of 1.72 ±0.28 mm and a mean echogenicity of 96.7. Increased vascularity was noted, with a mean PSV of 7.86 cm/sec. The resistive index was 1.01, indicating the presence of increased microvessel density.

## Conclusions

 UHFUS may be helpful in providing additional information to the clinical assessment of leukoplakia. Further investigation on larger samples is advised to correlate imaging findings with both clinical examination and histology.

# Abstract: Combining Curcumin and Paclitaxel to induce oral cancer cell death: an *in vitro study*

**Authors:** M. Rouffineau, J.P.N. Silva, H. Bousbaa, L. Delgado, S. Warnakulasuriya, P.M.A.. Silva, L. Monteiro

**Affiliations:** *UNIPRO—Oral Pathology and Rehabilitation Research Unit, University Institute of Health Sciences (IUCS), Cooperativa de Ensino Superior Politécnico e Universitário (CESPU),Gandra, Portugal. Email: leonordelgado@inno.pt*

## Objectives

 Treating patients with oral cancer remains challenging, despite advances in drug therapy and surgical techniques. Therefore, innovative therapeutic approaches are crucial. The main goal of this study is to explore the therapeutic potential of combining a natural compound namely Curcumin (Cur) with a chemotherapy drug Paclitaxel (PTX) to induce oral cancer cell death.

## Material and Methods

 The dose-response curves and IC50 of Cur and PTX for SCC-9 and SCC-25 oral cancer lines were determined using the MTT assay. A dual-cross concentration matrix evaluated the cytotoxic effects of combining Cur and PTX(Bliss Analysis). Annexin V-PI staining assessed cancer cell death by flow cytometry in Cur+PTX-treated cells. A colony formation assay evaluated the long-term proliferative effects of Cur+PTX.

## Results

 The combination of Cur+PTX demonstrated different effects on the viability of SCC-9 and SCC-25 oral cancer cells, showing synergistic effects on SCC-25 and antagonistic effects on SCC-9 cells. Cur+PTX enhances apoptotic cell death in SCC-25 cells and inhibits their long-term proliferation.

## Conclusions

 Combining Cur+PTX has therapeutic potential to induce oral cancer cell death in a manner dependent on the specific cell/cancer type, which deserves further exploration. This highlights the importance of considering personalized medicine when developing new therapeutic strategies for each patient.

# Abstract: Proteomic analysis of electronic cigarette users' saliva

**Authors:** N.C. Faria, B.F.C. Carvalho, M.G.O. Alves, I.F. Lima, L.K. Iwai, M.S. Salardani, A. Zelanis, J.D. Almeida

**Affiliations:** *Universidade Estadual Paulista (UNESP), Instituto de Ciência e Tecnologia, Campus de São José dos Campos, São Paulo, Brazil. Email: janete.almeida@unesp.br*

## Objectives

 The electronic cigarette (e-cig), has been widely used in recent years, especially by youngs. Cytotoxic and carcinogenic substances may be present in its composition; however, little is known about the risks of its use. The aim was to evaluate the protein profile of saliva from e-cig users through proteomic analysis and the possible salivary alterations.

## Material and Methods

 Two groups: Electronic Cigarette Group (ECG), composed of 25 regular and exclusive e-cig users, and Control Group (CG), consisting of 25 non-smokers and non-e-cig users, matched by sex and age to the ECG. All participants underwent clinical examination and unstimulated saliva collection for salivary flow rate measurement and evaluation of salivary proteome. The samples were subjected to the following analyses: salivary viscosity, pH, buffer capacity, and measurement of salivary cotinine performed using the ELISA Kit (Salimetrics). The alcohol consumption profile was assessed using the AUDIT. The e-cig consumption profile was traced through questions related to device usage and testing for carbon monoxide concentration in expired air. Proteomic analysis was performed using shotgun methodology and liquid chromatography coupled with mass spectrometry (LC-MS). Since the obtained results did not follow a normal distribution, they were subjected to the Mann-Whitney test.

## Results

 A higher concentration of exhaled CO was observed, along with a higher salivary concentration of cotinine, higher scores on the AUDIT, lower salivary viscosity, and an unfavorable result on oximetry. Regarding the salivary proteome, 48 proteins were specific to ECG, with 3 proteins being differentially expressed.

## Conclusions

 e-cig users have higher rates of alcohol consumption and lower salivary viscosity, important alterations in respiratory capacity demonstrated by increased CO concentration in expired air and decreased oximetry, and high levels of cotinine in saliva.

# Abstract: Differences between oral leukoplakia and proliferative verrucous leukoplakia. Study of genetic profiles

**Authors:** A. Díaz Rodríguez, M. Pérez-Sayáns, P. Gándara Vila, E. Otero Rey, A. Blanco Carrión, A. García García

**Affiliations:** *Department of Oral Medicine, Oral Surgery and Implantology. Faculty of Medicine and Dentistry. University of Santiago de Compostela. Email: alba.diaz.rodriguez0@rai.usc.es*

## Objectives

 To decipher the molecular mechanisms driving the origin and clinical behaviour of oral leukoplakia (OL) and proliferative verrucous leukoplakia (PVL), using RNA-Seq to analyse differential gene expression profiles and associated genetic variations.

## Material and Methods

 Patients with clinically and histopathological diagnosed LO or PVL were included in the study and 5x5mm incisional biopsies were taken and subjected to RNA-Seq.

## Results

 Polymorphisms and overexpressed genes were identified in PVL samples that could be considered as biomarkers. There are a large number of common or almost specific genetic variants for PVL. For the non-verrucous forms, there is a smaller number of variants, but almost all of them are grouped together.

## Conclusions

 At the genetic level, there are clear differences between oral leukoplakia and proliferative verrucous leukoplakia. There could be polymorphisms present in the non-verrucous forms that may point to their progression to PVL.

# Abstract: Multiclass classification of oral mucosal lesions by deep learning from clinical images

**Authors:** A. Redondo García, K. Ivaylova, M. Bachiller, M. Rincón, J.M. Cuadra, F. Tamini, J.L. López-Cedrún, M. Diniz-Freitas, L. Lago-Méndez, G. Rubín-Roger, J. Torres, L. Bagán, G. Hernández, R.M. López-Pintor

**Affiliations:** *ORALMED Research Group, Department of Dental Clinical Specialties, Complutense University of Madrid, Spain. Email: kateriva@ucm.es*

## Objectives

 Oral cancer is a frequently malignant tumour that can be detected during an oral examination. Unfortunately, it is often diagnosed in advanced stages. This study aims to investigate the effectiveness of using clinical images and deep learning-based models to perform a multiclass classification of oral lesions in photographs taken without restrictions.

## Material and Methods

 The classification differentiated four classes of oral lesions: malignant, potentially malignant, benign, and healthy. The dataset included a total of 3,246 images. The images show different areas of the oral cavity and were captured from various perspectives by different dentists and maxillofacial surgeons. Experiments have been conducted with networks encompassed within Convolutional Neural Networks and Recurrent Neural Networks, using different input image sizes. In addition, the region of interest (ROI) is used as input to the model.

## Results

 The best EfficientNet B0 model achieves an accuracy of 85.54%, precision of 84.09%, recall of 87.61%, F1-score of 85.52%, and ROC AUC of 95.65%.

## Conclusions

 This model improves on the proposals of previous studies by considering a larger number of oral lesions and output classes with promising results. It is necessary to assess whether the increase of the dataset can increase the accuracy of the proposed model.

# Abstract: Implications of p53 protein upregulation in oral lichen planus: a systematic review and metaanalysis

**Authors:** C. Keim del Pino, P. Ramos García, L.A. Pimenta Barros, M.A. González Moles

**Affiliations:** *School of Dentistry, University of Granada, Granada, Spain. Email: janete.almeida@unesp.br*

## Objectives

 The objective of this systematic review and meta-analysis was to qualitatively and quantitatively analyze the current evidence on the implications of p53 upregulation in oral lichen planus (OLP) assessed by immunohistochemical techniques, in order to identify molecular mechanisms involved in the behavior of OLP as an oral potentially malignant disorder (OPMD).

## Material and Methods

 We searched MEDLINE/PubMed, Embase, Web of Science and Scopus for studies published before February-2024. We critically assessed the methodological quality of primary-level studies and performed meta-analyses. Twenty five individual studies met the inclusion criteria, comprising 778 OLP samples, in which the expression of p53 was analyzed through immunohistochemistry.

## Results

 Most OLP displayed p53 protein upregulation (pooled proportion [PP]=66.26%, 95%CI=54.94-76.77). Regarding the magnitude of association analysis, oral squamous cell carcinoma (OSCC) cases showed a significantly higher frequency according to p53 expression in comparison to OLP (OR=2.79, 95%CI=1.84-4.24; p<0.001); while, OLP exhibited a significantly higher frequency for p53 expression in comparison to healthy controls (OR=6.48, 95%CI=3.17-13.24; p<0.001).

## Conclusions

 In conclusion, the present study demonstrates the frequent p53 protein upregulation, which is probably indicating an antitumor response in a epithelium whose cells are under cellular stress and at risk of cancer.

# Abstract: Oral cancer epidemiology is changing: what's happening and what are we doing?

**Authors:** M.C. Mateo-Sidrón Antón, R. Cerero Lapiedra, J.A. Celis Donner, L.A. Moreno López

**Affiliations:** *Department of Dental Clinical Specialties. Universidad Complutense de Madrid. Madrid. Email: mamate07@ucm.es*

## Objectives

 The aims of the study are to evaluate the ratio male/female and the correlation between number of dentists who practice in Madrid and the diagnostic stage of oropharyngeal cancer in Madrid.

## Material and Methods

 We use data from the Regional Cancer Registry of Cancer of Madrid published every year. We select data from 2014 to 2022 diagnosed with CIE code form C00-C14. Number of dentists were obtained from INE. A Spearman correlation was used to analyze the relation between diagnostic stage and the number of dentists in Madrid.

## Results

 There is a positive correlation between diagnostic stage and number of dentists, but we did not find statistical significance. There is a change in ratio M/F in this period, that could represent a change in risk factors and must be studied in every localization.

## Conclusions

 Despite the increase, in recent years, of cases of Oropharyngeal Cancer and registered dentists in the Community of Madrid, there’s still a delay in early detection of Oropharyngeal Cancer.

# Abstract: Increased plasmatic levels of exosomes are significantly related to relapse rate in patients with oral squamous cell carcinoma: a cohort study

**Authors:** B.J. Pinzón Rodríguez, S. Rodríguez Zorrilla, M. Pérez-Sayáns, A. Blanco Carrión, P. Gándara Vila, A. García García

**Affiliations:** *Oral Medicine, Oral Surgery and Implantology Unit (MedOralRes), Faculty of Medicine and Dentistry. Universidade de Santiago de Compostela. Email. babipinzon@gmail.com*

## Objectives

 This study aims to assess the exosomal profile as a potential tool for early detection of relapse and long-term outcomes in OSCC patients undergoing conventional therapy.

## Material and Methods

 27 OSCC patients with a median 38-month follow-up were included in this study. The relationship between NTA-derived parameters and clinical pathological parameters was examined, and receiver operating characteristic (ROC) curves were utilized to evaluate the diagnostic efficacy of these values in detecting cancer relapse.

## Results

 Plasmatic levels of exosomes prior to surgery showed a drastic reduction after surgical intervention. Postsurgical concentrations of exosomes were higher in patients who experienced relapse compared to those who remained disease-free. Additionally, patients who relapsed exhibited larger exosome sizes after surgery. Patients with lower concentrations of exosomes prior to surgery demonstrated better disease-free survival compared to those with higher levels (p = 0.012). ROC analysis revealed an area under the curve of 0.82 for presurgical exosome concentration in identifying relapse.

## Conclusions

 Presurgical exosomal plasmatic levels serve as independent predictors of early recurrence and survival in OSCC. All in all, our findings indicate that the detection of peripheral exosomes represents a novel tool for the clinical management of OSCC, with potential implications for prognosis assessment.

# Abstract: Prognostic and clinicopathological significance of epidermal growth factor receptor (EGFR) expression in oral squamous cell carcinoma: Systematic review and meta-analysis

**Authors:** V. Samayoa Descamps, J.L. Cívico Ortega, I. González-Ruiz, P. Ramos García, D. Cruz Granados, M.A. González Moles

**Affiliations:** *Faculty of Dentistry, University of Granada, Spain. Email: vasamayoa@correo.ugr.es*

## Objectives

 To evaluate the clinicopathological and prognostic significance of EGFR overexpression in oral squamous cell carcinoma (OSCC) through a systematic review and meta-analysis, using immunohistochemical techniques.

## Material and Methods

 We searched MEDLINE/PubMed, Embase, Web of Science, and Scopus for studies published before November 2022. The methodological quality of the studies was determined using the QUIPS tool (Cochrane Prognosis Methods Group). Meta-analyses were conducted, and heterogeneity was investigated through subgroup analyses and meta-regressions.

## Results

 Fifty primary-level studies (4631 patients) met the inclusion criteria. EGFR significantly associated with poor overall survival (HR=1.38; 95%CI=1.06-1.79; p=0.02) and N+ status (OR=1.37; 95%CI=1.01-1.86; p=0.04). More favorable results were obtained with a cutoff point ≥10% of tumor cells overexpressing EGFR (p<0.001).

## Conclusions

 EGFR overexpression in oral oncogenesis is associated with poor survival. Our results suggest immunohistochemical EGFR detection, using a cut-off point of 10% EGFR+ tumor cells to identify positive cases.

# Abstract: Salivary biomarkers and their potential use in the early diagnosis of oral cancer

**Authors:** I. Llinás, D. Vallejos

**Affiliations:** *ADEMA- University of Balearic Islands. Mallorca, Spain. Email: d.vallejos@eua.edu.es*

## Introduction

 Oral cancer has high morbidity and mortality rates and is the 16th most common malignant neoplasm worldwide. Early detection is crucial for a better prognosis. Saliva contains biomarkers that can be used for this purpose. Saliva is easy to collect and contains proteins that reflect health. The exposure of saliva to cancerous lesions makes it a specific and sensitive screening tool.

## Objectives

 To evaluate salivary biomarkers as a tool for early diagnosis of oral cancer, identifying limitations and feasibility as well as their impact on clinical practice.

## Material and Methods

 This literature review focused on studies published since 2017 on the use of salivary biomarkers in the diagnosis of oral cancer. A comprehensive search was conducted in PubMed and filters were applied to select the most relevant articles. Twenty-nine references were selected for analysis.

## Results

 MMPs, interleukins and TNF-alpha are important biomarkers in oral cancer. DNA and RNA can also be used as biomarkers in carcinoma detection. Metabolomics has identified more than 100 metabolites and proteins such as CD44 and CD63 as promising biomarkers in oral cancer progression, but limitations and other clinical factors must be considered for accurate assessment.

## Conclusions

 Salivary biomarkers have potential in the diagnosis of oral cancer but cannot replace biopsy and more studies and resources are needed to validate and standardize their clinical use.

# Abstract: Primary care interval in oral cancer: relative contribution to diagnostic delay by referring professionals - physicians and dentists. A systematic review and meta-analysis

**Authors:** P.I. Varela Centelles, J.M. Seoane Romero, N. Mallha, S. Warnakulasuriya, B. Takkouche

**Affiliations:** *CS Praza do Ferrol, Servizo Galego de Saúde. Lugo. Spain. Email: pabloignacio.varela@usc.es*

## Objectives

 Nearly half of oral cancer patients are diagnosed at an advanced stage. We aimed to quantify the primary care interval (PCI) in the diagnostic delay and to assess the impact of the referring physician’s speciality on the disease stage at diagnosis.

## Material and Methods

 We undertook a meta-analysis (protocol ID# CRD42022303426) on reports assessing oral/oropharyngeal carcinomas detailing starting- and endpoints for PCI, i.e. the time needed by the primary care physician to refer a patient suspected to have an oral cancer to a specialist, following the PRISMA guidelines. Quality assessment and publication bias were examined.

## Results

 The average PCI length was slightly larger for GP (30.5 days) than for GDP (27.6 days), while that for the total group was 28.7 days General practitioners (GP) consumed about a third of all the time elapsed since the first symptom to patient referral [PCI%: 0.31 (95% CI: 0.23, 0.40)]. General dental practitioners (GDP) refer their patients for treatment at earlier disease stages than GPs (Odds Ratio: 0.58; 95% CI: 0.34-0.98).

## Conclusions

 Primary care accounts for a considerable pre-hospital time for reaching a diagnosis of oral cancer patients, calling for measures to enhance early oral cancer symptom recognition in primary care settings. *Research Project Code: PI21/01206.

# Abstract: Gap in secondary care for patients with oral lesions

**Authors:** B.C. Dall’Orto Martins, L. Weinert de Freitas, A.C. Ferreira de Araujo, T. Duda Deps, F. de Azevedo Ribeiro, T.R. Grão Velloso, D.R. Camisasca, L.A.P. de Barros

**Affiliations:** *Graduate Program in Oral Sciences UFES. Brazil. Email: liliana.barros@ufes.br*

## Objectives

 Identify deficiencies in the oral healthcare network by mapping patients treated at an oral diagnostic service (ODS), correlating them with public secondary healthcare services(SHS).

## Material and Methods

 Cross-sectional study of secondary data analysis from the ODS, called Oral Diagnostic Center (ODC). Data from clinical records of patients with oral lesions referred from municipalities in the state of Espírito Santo were retrieved from 2011 to 2019. Patients were mapped by municipality(origin), correlated with the number of public SHS in the 3 macroregions of the state.

## Results

 The sample consisted of 1137 patients. The majority (75.28%) were from the Metropolitan region, the Central/North region with 11.35%, and the South region with 3.96%. Regarding SHS, the metropolitan region has 5 Dental Specialties Centers (DSCs), the Central/North with 2, and the South with 4. The Metropolitan region has the largest population, the largest number of DSCs and the largest flow to the ODC, while the Central/North region presents a significant percentage of ODC appointments associated with the reduced number of DSCs.

## Conclusions

 The deficiencies in the oral healthcare network are identified by the low number of DSCs, indicating weaknesses in SHS. Therefore, highlighting the contribution of ODS such the ODC in assistance patients with oral lesions.

# Abstract: OralImmunoAnalyser (OIA): a software for immunohistochemical assessment of oral leukoplakia using image segmentation and classification models

**Authors:** P. Akbari, M. Pena Cristóbal, V. Sanmartín Barragáns, J.M. Suárez Peñaranda, E. Cernadas, M. Gallas Torreira, P. Gandara Vila

**Affiliations:** *Grupo OralResMed. Universidad Santiago de Compostela, 2CITIUS. Universidad Santiago de Compostela. Email: pardis.akbari95@gmail.com*

## Objectives

 This study aims to present a software that allows the semi-automatic counting of stained cells by immunohistochemistry on an image.

## Material and Methods

 This software is freely available to researchers on the following page: https: //gitlab.citius.gal/analyser/oralimmunoanalyser. Oralimmunoanalyzer (OIA) allows one to select an area of interest, counts intracellular brown marks, and classifies it as high, low, or unstained. The count is saved in .csv format. OIA allows modification of the classification. In this study, the evaluation was carried out by comparing manual counting and OIA.

## Results and Conclusions

 The automatic processing of the images provided the following average performance: (1) the cell detection module achieved a sensitivity of 64.4%, specificity of 93%, and precision of 60.7%; and (2) the cell classification achieved an accuracy of 79.8%. OIA saves 56.5% of time spent by the traditional manual counting of cells, avoiding mistakes because the user can check at any time the cells counted.

# Abstract: Demographic and clinical analysis of oral squamous cell carcinoma in a tertiary hospital in Riyadh, Saudi Arabia

**Authors:** N. AlMadan, A.A. Alhamidi, B. Almalki, S. Aladhyani, M. Almohaya, N. Nkoyo Imelda, N. Uguru, A. Alrumeh

**Affiliations:** *Oral medicine and special care dentistry department, Prince Sultan Military Medical City, Riyadh, Saudi Arabia. Email: nmalmadan@gmail.com*

## Objectives

 To evaluate the clinical and demographic parameters of the patients diagnosed with oral squamous cell carcinoma and compare disease outcomes of the patients who used Shammah (smokeless tobacco) with patients who didn’t use it.

## Material and Methods

 Retrospective single-center study on Prince Sultan Military Medical City (PSMMC) from 2010 to 2022. Inclusion criteria include patients who were diagnosed with OSCC, while the exclusion criteria include: 1) Treated outside our institute, 2) No follow-up data, 3) There is no histopathological slide in our pathology archives. Clinical records for patients were reviewed. A total of 192 cases were retrieved, but only 89 cases met our inclusion criteria.

## Results

 We identified 89 cases with male predilection (59.6%) with a mean age of 61.9 (SD: 15.58), with most of the cases diagnosed in the lateral tongue (61.8%). Pain was the first symptom of 37 cases, and 34 cases presented clinically with an ulceration. Shammah and tobacco smoking were reported in 21 and 14 patients, respectively. In 23 cases (25.8%), recurrence was reported, while 30 cases (33.7%) died because of the disease. Older age was related to more death cases. Correlation analysis and linear regression showed no significant difference in survival outcomes of patients who used Shammah, tobacco smoking, and the combination of both with a P value greater than 0.05.

## Conclusions

 OSCCs in PSMMC have high mortality rates, with older patients reporting worse disease outcomes. However, no correlation between shammah use and OSCC outcomes was demonstrated.

# Abstract: Assessing accuracy of diagnostic capabilities of A.I. in oral and maxillofacial pathology

**Authors:** M. Cuevas Nuñez, V. Alvarez Silberberg, M.J. Biosca Gómez de Tejada, R. Posada, R. Ballester Victoria, I. Koptseva, B.C. Jham, J. Bara Casaus, M.T. Fernández Figueras

**Affiliations:** *Universitat International de Catalunya. Spain. Email: mcuevasnunez@uic.es*

## Objectives

 The Chat Generative Pretrained Transformer (ChatGPT) is a digital platform of artificial intelligence (AI) that uses deep learning to generate responses to questions in a manner similar to human interactions. This study compared the diagnostic capabilities of ChatGPT-3 with the histopathological diagnoses of pathologists.

## Material and Methods

 Anonymized clinical and histopathological data from 36 cases (n=36) referred to the pathology department of the General University Hospital of Catalonia between January 2022 and April 2024 were transcribed into ChatGPT-3, followed by the question, "What is the diagnosis?" The diagnosis suggested by ChatGPT-3 was compared with the diagnosis made by pathologists.

## Results

 Upon transcribing information about gender, age, site, and histopathological description of the lesions, ChatGPT-3 made accurate diagnoses in 44% of cases (n=16). ChatGPT-3 gave differential diagnoses for all cases (100%, n=36). In all oral dysplastic lesions (100%), AI was unable to determine the grade. Interestingly, when the affected site was included, ChatGPT-3 made more errors compared to when the affected site was not included.

## Conclusions

 Altogether, when more information was given to ChatGPT-3, more errors were seen in the diagnoses. ChatGPT-3 is not able to apply clinical diagnostic reasoning skills like trained professional human analyses.

# Abstract: Analysis of the main epidemiological and prognostic characteristics of oral squamous cell carcinoma in the Basque Autonomous Community (period 2012-2017)

**Authors:** I. Amezaga Fernández, I. Lafuente Ibáñez de Mendoza, X. Marichalar Mendia, A. López de Munain Marques, J.M. Aguirre Urizar

**Affiliations:** *Department of Stomatology. Faculty of Medicine and Nursery, University of the Basque Country (UPV/EHU). Leioa, Spain. Research Group GUI21/042. Email: iamezagafer@gmail.com*

## Objectives

 To describe the main epidemiological and prognostic characteristics of oral squamous cell carcinoma (OSCC) in the population of the Basque Autonomous Community (CAPV), from 2012 to 2017.

## Material and Methods

 This is a prospective observational study, analysing data on patients diagnosed with oral cancer between 2012 and 2017, obtained from the Basque Cancer Registry. A descriptive and survival analysis with Kaplan Meier curves was carried out with the variables collected.

## Results

 1631 patients were diagnosed, 29.6% being women and 70.4% men, with a mean age of 66.96 years (SD±12.34). Crude incidence rates were 18.1 for men and 7.2 for women, per 100 000. Most of the patients (57.6%) resided in Bizkaia, and 91.7% lived in urban settings. Conventional OSCCs accounted for 95.6%, and the tongue (30.9%) was the most common location. At diagnosis, 52% of cases were disseminated. 5-year overall and disease-specific survival were 50.2% and 57.8%, respectively.

## Conclusions

 OSCC in the CAPV continues to be more common in older men, urban residents and in the tongue. It still has a poor prognosis, with many cases diagnosed in advanced stages. It is necessary to implement preventive and early diagnostic protocols in the Basque Country for this pathology.

# Abstract: Association of autoimmune disorders and malignant transformation in oral lichen planus: A systematic review and meta-analysis

**Authors:** L. Bagan, A. Proaño Haro, M. Margaix Muñoz, A. Rubert, M.G. Sarrion Perez, J. Bagan

**Affiliations:** *Department of Stomatology, Oral Medicine. University of Valencia, Valencia, Spain. Email: leticia.bagan@uv.es*

## Introduction

 Oral lichen planus (OLP) is a chronic inflammatory disease of autoimmune origin that has been related to various autoimmune pathologies, although its true etiology is unknown. The objectives of the present systematic review and meta-analysis are to assess the prevalence of autoimmune diseases in patients with the prevalence of autoimmune diseases in patients with OLP and its possible relationship with malignant transformation (MT).

## Material and Methods

 An electronic bibliographic search was performed in the PubMed, Embase and WOS databases until February 2024. The methodological quality of the studies, meta-analysis and analysis of heterogeneity and sensitivity. Publication bias was analyzed using forest plots.

## Results and Discussion

: From the initial pool of 459 studies, 31 articles were deemed relevant and included in the analysis. A total of 6596 OLP patients were studied, revealing a significantly higher relative frequency of autoimmune diseases (OR = 2.167, 95% CI: 1.32-3.57, p=0.002). Notably, a high prevalence of diabetes (PP=11.2%, 95% CI: 8.9-139) and thyroid disease (PP=15%, 95% CI: 8.3-25.6) was observed in OLP patients. These findings underscore the high prevalence of autoimmune diseases in OLP patients (PP=11.2%, 95% CI: 8.9-139), a crucial aspect for the medical and research community to consider. relative frequency of autoimmune diseases (OR = 2.167. 95% CI: 1.32-3.57. p=0.002). A high prevalence of prevalence of diabetes (PP=11.2%. CI 95%: 8.9-13.9) and thyroid disease (PP=15%. 95% CI: 8.3-25.6) in the OLP. The prevalence of autoimmune diseases in patients with OLP who developed MT in OSCC was 29.3% (CI 95%: 17-45.7).

# Abstract: Oral lichen planus – a treatment challenge

**Authors:** M. Balcheva

**Affiliations:** *Department of Conservative dentistry and oral pathology, Faculty of Dental Medicine, Medical University of Varna. Bulgaria. Email miglena.balcheva@mu-varna.bg*

## Objectives

 To present the difficulty in achieving a sustainable result in the treatment of oral lichen planus.

## Material and Methods

 The survey includes patients from both sexes and all age group who visited our Faculty in the period 2011 – 2024. Their lesions were diagnosed clinically and/or histologically; in addition, patch tests with dental haptens were applied to confirm or exclude an allergic etiology. Patients were treated with corticosteroids or tacrolimus, and results were monitored.

## Results

 We have diagnosed 115 patients with oral lichen planus. They are with average age of 56.69±12.48 years. Females prevail significantly. In 60% of cases diagnosis was confirmed histologically, as reticular form was the most common one, and buccal mucosa was the most frequent site (44.9%). Allergy to dental materials was confirmed in 37.3% of the studied patients. We usually treated patients with topical steroids, and recurrence was registered in 29.6% of them. There were no cases of malignant transformation.

## Conclusions

 Our results confirm the imperfection of the lichen planus therapy, but also the negligible risk of malignancy.

# Abstract: Profile of oral squamous cell carcinomas at the Pathological Anatomy Laboratory of the School of Dentistry of Universidad de la República, 1982-2023 period

**Authors:** V. Beovide, N. González, V. Tancredi

**Affiliations:** *Pathology and Oral Medicine Departament, Pathological Anatomy Laboratory Subunit. School of Dentristy, Universidad de la República. Montevideo, Uruguay. Email: beovide@gmail.com*

## Objectives

 Determine the profile of oral squamous cell carcinoma (OSCC), in the Anatomic Pathological Laboratory of the School of Dentistry of the Universidad de la República, in the period 1982-2023.

## Material and Methods

 A retrospective, cross-sectional and analytical study was carried out. 252 patients with OSCC were studied during the period 1982-2023. Descriptive statistical analysis and survival curves (Kaplan-Meier) were performed.

## Results

 The age range was from 16 to 99 years, with a mean of 63 years ± 12.57. The 55,5% were male, with an M:F ratio of 1.25:1. The 44% were well differentiated, 46%, moderately differentiated and 9,6% undifferentiated. The most common location was the alveolar ridge mucosa (29,2%), the tongue (24,8%). The mean survival time was 2.36 years (CI: 1.60 – 8.24) and the 5-year survival rate, without considering the disease stage, was 43%.

## Conclusions

 OSCC continues to be a pathology with a low survival rate. This study allowed to learn more about this entity, in a reference service in oral pathology at the country level and generate our own data for subsequent research studies.

# Abstract: Meta-analysis study and sequential analysis of trials on Tumor Budding as a prognostic factor in head and neck squamous cell carcinomas

**Authors:** R. Cáceres Pinto, F.F.V. e Silva, V.C.A. Caponio, M. Pérez-Sayans, M.E. Padín-Iruegas, A. Blanco Carrión

**Affiliations:** *Department of Surgery and Medical-Surgical Specialities. Faculty of Medicine and Dentistry. Universidad de Santiago de Compostela. Spain. Email rodrigo.acap99@gmail.com*

## Objectives

 Head and neck cancer represented a growing number, compared to previous years, of approximately 750 thousand new cases, with the region of the lips and oral cavity being the one with the highest number of deaths. Due to the heterogeneity in survival within the same tumor stages, there is a need for new additional biomarkers, such as tumor budding. For this reason, a specific study was carried out on the relationship between a worse prognosis in head and neck squamous cell carcinomas and tumor budding.

## Material and Methods

 A comprehensive meta-analysis was performed to investigate the relationship between tumor budding and squamous cell carcinoma and related outcomes.

## Results

 In total 42 studies were included, patients with high levels of tumor budding reported an overall survival (OS), hazard ratio (HR) of 2.63, disease-free survival (DFS) HR of 1.88 and disease-specific survival. (DSS) of 2.14

## Discussion

 The presence of tumor gemination was strongly associated with worse survival in patients with head and neck squamous cell carcinoma, with HR of 2.63 (2.04-3.39; p<0.001), HR of 1.88 (1.57-2.24; p< 0.001) and HR of 2.14 (1.81-2.52; p<0.001), in reference to OS, DFS and DSS respectively.

## Conclusions

 The consistent findings analyzed in this study specifically indicate that tumor budding may be useful as a histological prognostic marker for HNSCC, however, more controlled trials are necessary.

# Abstract: Clinical study of 287 patients with oral lichen planus and its evolution

**Authors:** L. Campo Fernández, J.M. Somoza Martín, P. Gándara Vila, E. Otero Rey, A. Blanco Carrión

**Affiliations:** *Máster Medicina Oral, Cirugía Oral e Implantología. Universidad de Santiago de Compostela. Email: laucampofernandez@gmail.com*

## Objectives

 To study the evolution of patients diagnosed clinically and histologically with oral lichen planus and lichenoid lesions.

## Material and Methods

 A review of patients with oral lichen planus (OLP) followed from the Oral Medicine Unit of the USC was carried out. Starting from 588 patients, the inclusion and exclusion criteria were applied and the final sample was 287 patients.

## Results

 Of these 287 patients, 71.3% were women. White lichen planus predominated (72%), so the majority of patients are asymptomatic. The most frequent location, excluding multiple location, was the buccal mucosa, with a significant difference. Regarding the evolution, more than 90% of the patients continued to present OLP. However, 6.27% progressed to proliferative verrucous leukoplakia (PVL) and 1.04% to oral cancer. Of the 18 patients who developed PVL, the majority were women (87%), half were non-smokers. It is interesting to note that ¾ of these patients who developed PVL had a history of lichenoid lesions. OLP patients who progressed to oral cancer were 3, of which 2 were women and none had any toxic habits.

## Conclusions

 OLP is a chronic, changing inflammatory disease that is part of the potentially malignant oral lesions. Therefore, the continuous review of our patients is crucial to detect possible changes in the lesions.

# Abstract: Knowledge, attitudes and practices about oral pathologies in an adult population of Gran Canaria

**Authors:** J. Cassol Spanemberg, S. Parra Rojas, C. Molina Rodríguez, F. Raynal Floriano

**Affiliations:** *Facultad de Ciencias de la Salud. Departamento de Odontología. Universidad Fernando Pessoa Canarias. Email: jcassol@ufpcanarias.es *

## Objectives

 To ascertain the level of knowledge, practices, and attitudes regarding oral pathologies, with a focus on oral cancer, among patients at the University Fernando Pessoa Canarias Dental Clinic.

## Material and Methods

 A quantitative, descriptive, cross-sectional study was conducted. Data were collected using an assessment instrument. The study included 101 adult patients, both men and women. Statistical analysis was performed using Stata v.16 software.

## Results

 To our knowledge, this is the first study in the Canarian population and the findings indicate that the knowledge, attitudes, and practices of the studied population in the Canary Islands are insufficient and inadequate. The results reveal widespread ignorance regarding oral cancer and its etiology, risk factors, signs and symptoms, as well as general oral pathologies and appropriate actions or referrals for oral lesions.

## Conclusions

 It is crucial to implement health education campaigns targeting the population on oral pathologies, with a special focus on oral cancer. A strategy to address this challenge includes executing specific public education programs for both young and adult populations, aimed at enhancing knowledge and awareness of oral cancer, potentially leading to earlier detection and improved treatment outcomes.

# Abstract: Something is changing in gingival cancer. Evolution from 2001 to 2022 based on CMBD data

**Authors:** J. Celis Dooner, M.C. Mateo-Sidrón Antón, R. Cerero Lapiedra, L.A. Moreno López

**Affiliations:** *Universidad de los Andes, Santiago, Chile. Email: jocelis@ucm.es*

## Objectives

 To describe the epidemiological evolution of gingival cancer in the Spanish population from 2001 and 2022.

## Material and Methods

 A cross-sectional observational study was carried out from May to June 2024, using the databases obtained from the "Conjunto Mínimo Básico de Datos – Hospitalización" (CMBD-H) and the "Registro de Atención Especializada" (RAE-CMBD). The variables recorded included sex, age, lesion location, smoking, and alcohol consumption. The age-adjusted incidence was calculated."

## Results

 5,749 patients were included, 2,978 (51.8%) were men and 2,771 were women (48.2%), with an overall mean age of 68.3 years (SD ± 13), 65.55 years (SD ± 12.73) in men and 71.26 years (SD ± 12.66) in women. There was an observed inversion in the male/female ratio with a final predominance in women. An age-adjusted incidence of 0.60 will be observed in the overall population: 0.69 in men and 0.52 in women.

## Conclusions

 There is a change in the epidemiological distribution of gingival cancer, characterized by an increase in the age of onset, a predominance in women and a disconnection with known risk factors.

# Abstract: Gardner Syndrome and oral manifestations: A report of two clinical cases

**Authors:** R. Cerqueira, S. Ferreira, L. Delgado, F. Salazar, J.J. Pacheco, S. Warnakulasuriya, L. Monteiro

**Affiliations:** *UNIPRO—Oral Pathology and Rehabilitation Research Unit, University Institute of Health Sciences (IUCS), Cooperativa de Ensino Superior Politécnico e Universitário (CESPU), Gandra, Portugal. Email: ritateixeiracerqueira@gmail.com*

## Objectives

 Our aim is to present and discuss the clinical presentations of two cases of Gardner syndrome (GS).

## Case Report

 Case1: We present a case of a 47-year-old man who presented with an asymptomatic mass on the mandible. During anamnesis, he revealed a history of intestinal polyposis (IP) manifestations and had a colectomy at age of 20 years but was not aware of GS diagnosis. He had a family history of IP (mother and 2 brothers). On clinical and radiological imaging examination we identified multiple osteomas of the right mandible and he had several epidermoid cysts on his skin. Diagnosis of GS with confirmation of APC gene mutations in chromosome 5q22 was made. Case 2: Other case was seen in 60 years-old women with a mandibular osteomas. Anamnesis revealed familial adenomatous polyposis, and had been submitted to colectomy. On clinical and radiological imaging we identified multiple osteomas of the mandible and skull, tooth impaction, and dental abnormalities, all compatible with the manifestations of the GS disease.

## Conclusions

 The characteristics of Gardner Syndrome are important for potential diagnosis of these patients during oral health consultations not only for the treatment of oral / mouth lesions in these patients but also because they could be important in the early diagnosis of Gardner syndrome.

# Abstract: Recent trends in oral cancer risk factors and clinical characteristics among women: A systematic review

**Authors:** N. Dar-Odeh, D. Arabiat, S. Abu-Hammad, D. Fatafteh, O. Abu-Hammad

**Affiliations:** *School of Dentistry, The University of Jordan, Jordan School of Nursing, The University of Jordan, Jordan. Email: najla_dar_odeh@yahoo.com*

## Objectives

 To identify demographic and clinical characteristics of oral cancer (OC) affecting women, and to explore associated potential risk factors in the past twenty years.

## Material and Methods

 We systematically reviewed the literature in three databases to retrieve case reports published during the period 2000-2024 that described OC in women. Retrieved data included age, risk factors, clinical presentation and delay in diagnosis.

## Results

 A total of 56 case reports/ series were included with a total of 64 cases described. A proportion of 32.8% was reported from East Asia. Mean age was 48.7±19.9 years (17-88 years). A proportion of 89.6% constituted SCC in the tongue (47.4%) presenting mainly as a lump (39.1%), and ulcer (35.9%). The prevalence of tobacco use was 25%, and alcohol use was (7.8). 39 cases reported a delay in diagnosis (two weeks - eight years) with an average of 9.5 months.

## Conclusions

 Most cases of OC among women were SCC and originated from East Asia. Traditional risk factors were not identified in most cases. An average duration of 9 months of delay in diagnosis was identified. Improvements are warranted in healthcare systems particularly in low resource settings to spread awareness among patients, their families and their healthcare providers.

# Abstract: Hallmarks of cancer biomarkers in Proliferative Verrucous Leukoplakia: A scoping review

**Authors:** A. Proaño Haro, L. Bagan, A. Rubert, Y. Jimenez, M. Margaix Muñoz, J. Bagan

**Affiliations:** *Department of Stomatology, Oral medicine, University of Valencia, Valencia, Spain. Email: alex_dx2@hotmail.com*

## Objectives

 This scoping review examines the current state of evidence – based knowledge on altered molecular mechanisms in PVL according to the hallmaks of cancer, identifies gaps in evidence and suggests future research directions.

## Material and Methods

 This study was carried out following PRISMA-ScR statement guidelines. We included studies evaluating the lastest update of hallmarks of cancer published in 2022, with no date or language restrictions. Medline (PubMed), EMBASE, and Web of Science electronic databases were searched. The search strategy was based on the following keywords (MeSH, EMTREE terms): "Proliferative verrucous leukoplakia" in conjunction with biomarkers or oncogenic related processes.

## Results

 Of the 923 identified studies, 27 were found to meet the inclusion criteria. The hallmarks that have been most extensively studied were "genome instability & mutation" (n=9), "evading growth suppressors" (n=7) and "sustaining proliferative signaling" (n=6). There were limited scientific evidence and many gaps for several hallmarks of cancer and a complete lack in areas such as "resistance to cell death", "enabling replicative immortality", "inducing angiogenesis", "deregulating cellular metabolism" and "unlocking phenotypic plasticity".

## Conclusions

 Despite the increase in scientific publications in recent years, more primary – level studies are required to fully understand the molecular pathogenesis of PVL, which is still not completely characterised.

# Abstract: Clinical aspects of gingival carcinoma: a bibliographic review

**Authors:** J. Epelde Eizaguirre, I. Lafuente-Ibáñez de Mendoza, J.M. Aguirre Urizar

**Affiliations:** *Department of Stomatology. University of Basque ntry/EHU. E-mail: jepelde.azkoitia@gmail.com*

## Objectives

 To review and analyze the main clinical and demographical aspects of gingival squamous cell carcinoma and compare them with other carcinomas located in different sites of the oral cavity.

## Material and Methods

 A bibliographical search of the PubMed, Web of Science and Scopus databases was conducted. The research was made by the combination of different entry keyword terms: gingiva, gum, "oral cancer" and "oral squamous cell carcinoma". We only selected studies on humans, including retrospective case-controls, case-series and cohort studies, published since 2019.

## Results

 In total, 2012 records were retrieved but only 16 articles were selected for the review. The prevalence of gingival carcinoma was similar in men and women, primarily affecting non-smokers and non-drinkers, and was frequently diagnosed in advanced stages. Overall, SCC of other intraoral sites were mainly diagnosed in advanced stages and in men over 60 years with heavy tobacco and alcohol intake; being mainly located in the tongue.

## Conclusions

 Clinical characteristics of gingival squamous cell carcinoma, including patient sex and age, risk factors and tumor size, are similar to other carcinomas of the oral cavity, but further research is needed to better understand differences in prognosis of these patients.

# Abstract: m-Health to enhance oral cancer awareness in older adults in Chile: a preliminary report

**Authors:** C. Morales, I. Espinoza, R. Leonardo, V. Beltrán

**Affiliations:** *Facultad de Odontología de la Universidad de Chile.Email: iespinoza@odontologia.uchile.cl*

## Objectives

 This study aims to assess a newly developed mobile app's efficacy in raising oral cancer awareness among older adults through serious gaming integration.

## Material and Methods

 A novel application named TEGO® (Tele-platform of Geriatric and Dental Specialties in Spanish) was developed, showcasing a validated video on oral cancer and its risk factors assessed with the Patient Education Materials Assessment Tool (PEMAT). Additionally, serious gaming elements like trivia and word search puzzles were integrated. Sixty patients, aged 60 to 70 years, were randomly selected from the patient pool at the Dental Clinic of the University of Chile. Pre- and post-intervention questionnaires evaluating knowledge and attitudes towards oral cancer were administered.

## Results

 Initial findings reveal a participation rate of 45.1% among the invited individuals, with a majority being male (62.5%). Among the participants, 37.5% demonstrated prior awareness of oral cancer, yet none had engaged in oral self-examinations. Following one week of app usage, there was notable engagement, with 100% of participants utilizing the application, leading to positive alterations in knowledge.

## Conclusions

 The utilization of mHealth technology exhibits potential as an effective educational tool for disseminating knowledge about oral cancer among older adults.

# Abstract: Treatment of oral lichen planus with CO2 laser: a randomized clinical trial with preliminary results

**Authors:** L. Goce, B. Alvarez, S. Ferreira, A. Guida, S. Warnakulasuriya, L. Monteiro

**Affiliations:** *UNIPRO—Oral Pathology and Rehabilitation Research Unit, University Institute of Health Sciences (IUCS), Cooperativa de Ensino Superior Politécnico e Universitário (CESPU),Gandra, Portugal. Email: sarasousaferreira.md@gmail.com*

## Objectives

 To evaluate the effectiveness of vaporization with CO2 LASER of OLP lesions, in reducing pain and improving clinical appearance.

## Material and Methods

 Prospective randomized clinical trial. Patients (7 females and one male) with symptomatic bilateral OLP in the buccal mucosa were included. Randomly, one side was treated with CO2 LASER (3-5W, continuous mode) using the vaporization method (intervention group) and the opposite side underwent clinical follow-up (control group). Symptoms and clinical appearance were assessed using the VAS scale, the reticular-erythema-ulcerative (REU) and Thongprasom sign (TSS) scales before the intervention and 1 month after the intervention.

## Results

 Preliminary results point to an improvement in REU and TSS records for the side of the mouth treated with laser between the beginning and the first follow-up, without side effects. Furthermore, there was a reduction in the VAS scale score (initial mean = 7.7 vs mean final follow-up = 2.3), suggesting that CO2 laser vaporization is a useful intervention for reducing symptoms of OLP.

## Conclusions

 OLP vaporization with the CO2 laser seems an effective treatment to reduce symptoms without any side effects and with an improvement of clinical appearance of the disease.

# Abstract: Prognostic analysis of peripheral blood values in oral squamous cell carcinoma in Galicia

**Authors:** A. García García, I. Amezaga Fernández, I. Lafuente-Ibáñez de Mendoza, X. Marichalar Mendia, A. Blanco Carrión, J.M. Aguirre Urizar

**Affiliations:** *Department of Stomatology. Faculty of Medicine and Nursery, University of the Basque Country (UPV/EHU). Leioa, Spain. Research Group GUI21/042. Email: iamezagafer@gmail.com.*

## Objectives

 To analyse the relation between the general values from peripheral blood tests and survival of oral squamous cell carcinoma (OSCC) in Galicia.

## Material and Methods

 We retrospectively analysed 229 cases of OSCC diagnosed and treated at Santiago de Compostela University Hospital Complex between 2010-2015. Epidemiological, clinical and evolutionary data were collected, as well as hemoglobin, red blood cell, platelet, lymphocyte, neutrophil and monocyte counts at the time of OSCC diagnosis. Neutrophil-to-Lymphocyte Ratio (NLR) and Systemic Inflammatory Response Index (SIRI) were calculated. For survival analysis, Kaplan-Meyer curves and the Cox regression model were used.

## Results

 In univariate analysis, the following were significantly related to overall and disease-specific survival: hemoglobin, red blood cell count, hematocrit, platelet count, neutrophil count, NLR and SIRI. Red blood cell and platelet counts, NLR and hematocrit levels were statistically significant in multivariate analysis.

## Conclusions

 Several peripheral blood values (red blood cells, haematocrit, platelets and NLR) are associated to the prognosis of OSCC patients in Galicia. Therefore, they should be considered in the pre-therapeutic oncological assessment of patients with OSCC.

# Abstract: Local antibiotherapy in oral biopsy: a transversal study

**Authors:** M.F. García Luna, M.J. Lucero Berdugo, F. Molina Miñano, A. Ferrández Pujante, C. Castillo Felipe, R. Bello Sánchez, J.F. Martínez Lage-Azorín, L. Párraga Linares, M. Cobos Martín

**Affiliations:** *Facultad de Ciencias de la Salud. Universidad Catoìlica San Antonio de Murcia (UCAM). Email: fmolina@ucam.edu*

## Introduction

 Oral biopsy is a minor surgical procedure and histopathological evaluation is essential for a definitive diagnosis in oral cavity lesions. Oral cavity wounds are considered as clean/contaminated and the classic protocol after biopsy is systemic antibiotic therapy.

## Objectives

 to evaluate the efficacy of local antibiotic therapy (Gelcide®) in the prevention of infections in oral biopsies.

## Material and Methods

 The design of this study was quasi experimental with a non-equivalent control group. In one of the groups, Gelcide® was applied after the biopsy procedure, and in the control group, systemic antibiotic therapy was prescribed.

## Results

 The sample of participants in this study consisted of 15 patients aged between 22 and 81 years. A Mann-Whitney U analysis was performed to measure pain and inflammation where there were significant differences in pain and no significant differences were observed in inflammation. A Chi-square test was performed where we obtained 100% efficacy in bleeding.

## Conclusions

 Gelcide® has proved to be a highly effective product to prevent possible infections in oral biopsies as well as pain, bleeding and scarring. However, we have not obtained significant efficacy results for the reduction of inflammation.

# Abstract: Photobiomodulation in the treatment of oral mucositis in oncological patients

**Authors:** M.E. González Moreno, I. Gallardo Castillo, J. Caballero Aguilar, M.J. Cobos Fuentes, Á. Martínez-Sahuquillo Márquez

**Affiliations:** *Facultad de Odontología. Universidad de Sevilla. España. Email: elgomo71aep010203@hotmail.com*

## Objectives

 The prevalence of oral mucositis induced by head and neck radiotherapy is 80% of treated patients Reducing the severity and duration of oral mucositis in patients who have undergone radiotherapy and chemotherapy will have a sidnificant impact on morbidity and mortality, as well as the cost of necessary care. Photobiomodulacion has proven to be a complementary treatment alternative to achieve analgesic, anti-inflamatory and biostimulation effects that promote tissue repair. The objetive of this work is to conduct a review of the available scientific literature regarding photobiomodulation for the treatment of oral mucositis caused by radio or chemotherapy.

## Material and Methods

 A search of articles was carried out in the following databases: Scopus, Pubmed and Cochrane between January 2019 and January 2024.

## Results

 When light is absorbed by the body, the energy that light has is converted into cellular energy, starting a set of metabolic events, which induce analgesia, regeneration, or anti-inflammatory effect.

## Conclusions

 PBM reduce the prevalence of more severe grade of OM induced by chemoradiotherapy. Also, PBM therapy reduce the duration of severe OM and improve QoL.

# Abstract: Leukocyte and platelet rich fibrin in the management of medication-related osteonecrosis of the jaw: A systematic review and meta-analysis

**Authors:** A. González Olazabal, A. Lorenzo Pouso, A. Muñoz Salgado, A. Gándara Vila, M. Pérez-Sayáns, A. Blanco Carrión

**Affiliations:** *Oral Medicine, Oral Surgery and Implantology Unit (MedOralRes), Faculty of Medicine and Dentistry, University of Santiago de Compostela, Santiago de Compostela, Spain. Email: aitorodontologia@gmail.com*

## Objectives

 To analyze the results obtained in the literature with the use of L-PRF in the treatment of ONJ through a systematic review and meta-analysis.

## Material and Methods

 Medline (via PubMed), Cochrane, Web of Science and Grey Literature Database was screened from which 10 were selected.

## Results

 In the meta-analysis with full resolution, combining the use of L-PRF in the treatment of ONJ, a weighted proportion of 94.3% of complete resolution is obtained, with a low degree of heterogeneity, statistically significant. When analyzing the non-resolution data, a weighted proportion of 7.7% was obtained with moderate heterogeneity. In the meta-regression, no significant correlation was found between complete resolution and year of publication. In consistency analysis no major changes in weighted proportion are identified when any of the studies are eliminated, demonstrating a high reliability in the combined results.

## Conclusions

 L-PRF alone or in combination with other therapies in treatment of ONJ achieved high percentages of complete lesion resolution (94.3%). In studies where L-PRF is combined with other therapies, and where the effectiveness of the other therapy alone is analyzed, L-PRF has been shown higher percentages of resolution.

# Abstract: Narrow Band Imaging (NBI) for early diagnosis of oral squamous cell carcinoma (OSCC): present and future

**Authors:** A. Guida, L. Monteiro, S. Tovaru, S. Warnakulasuryia

**Affiliations:** *A.O.R.N. "A. Cardarelli", U.O.C. Odontostomatologia, Naples, Italy. Email: agostino.guida@aocardarelli.it*

## Objectives

 Five-year survival of Oral Squamous Cell Carcinoma (OSCC) is around 60% and has not improved during the last decades, partly due to diagnostic delay. Narrow Band Imaging (NBI) fibroscopy seems to facilitate early diagnosis, using blue/green light to reveal capillary vasculature within the superficial mucosa and submucosal regions. There is no definitive scientific evidence that NBI is superior to oral examination with white light (WLOE) or other adjunctive aids for diagnosing OSCC or Oral Potentially Malignant Disorders (OPMDs).

## Material and Methods

 Current scientific literature will be reviewed and clinical cases will be presented.

## Results

 NBI has the ability to discern between malignant, pre-malignant and healthy tissue (sensitivity/specificity: 97% / 89%1). It may help with follow-up of chronic diseases (e.g. Oral Lichen Planus) and in the decision process of biopsy site of extended/multiple lesions.

## Conclusions

 Giving these promising results, a multicentre prospective randomised study has been designed, including patients with: 1) Oral mucosal lesions necessitating diagnosis; 2) OPMDs; 3) history of Head & Neck SCC; 4) high risk conditions (heavy smokers/drinkers, Fanconi anaemia, etc…) without oral lesions. Primary outcome will be a comparison of WLOE vs. NBI detection rates of OSCC/OPMD. Secondary outcome will be to estimate overall diagnostic accuracy of the two methods.

# Abstract: Assessment of depth of invasion in oral squamous cell carcinoma with ultra-high frequency ultrasonography: prospective cohort study

**Authors:** R. Izzetti, P. de Francesco, F. Minuti, M. Nisi

**Affiliations:** *University of Pisa, Department of Surgical, Medical and Molecular pathology and Critical Care Medicine, Pisa, Italy. Email rossana.izzetti@unipi.it*

## Objectives

 The aim of this study was to assess the reliability of intraoral ultra-high frequency ultrasonography (UHFUS) in estimating pre-operative depth of invasion (DOI) and Worst Pattern of Invasion (WPOI) with respect to histology in patients aNected by oral squamous cell carcinoma (OSCC).

## Material and Methods

 Consecutive patients with tongue OSCC were enrolled and underwent UHFUS examination (ultrasound frequency 48 MHz). The patients were scheduled for surgery, and histopathological DOI and WPOI were recorded. UHFUS DOI and cohesive/non-cohesive WPOI were assessed and compared to histology. OSCC lesions were stratified depending on their dimensions (DOI <5 mm; DOI 5-10 mm; DOI >10 mm).

## Results

 Forty-five patients (mean age 63.3±15.2 years) were enrolled. Mean DOI was 8.2±4.5 mm. Cohesive pattern of invasion was found in 11 cases and corresponded to WPOI 1-3 in 78.3% of cases. Non-coesive WPOI corresponded to a discontinuous pattern of invasion in 82.6% of cases. A significant correlation between ultrasonographic DOI and histology was found for tumours ≤ 10 mm (p<0.05).

## Conclusions

 UHFUS is a reliable tool for the prediction of tongue OSCC invasiveness due to the high consistency between imaging and histology, and may be a useful tool in the diagnostic work-up and surgical management of OSCC.

# Abstract: Differential histopathological features of gingival squamous cell carcinoma: a literature review

**Authors:** I. Lafuente-Ibáñez de Mendoza, E. Pérez Campo, X. Marichalar Mendia, J.M. Aguirre Urizar

**Affiliations:** *Department of Stomatology. University of Basque Country/EHU. E-mail: Irene.lafuente@ehu.eus*

## Objectives

 To update the histopathological differential aspects of squamous cell carcinoma of the gingiva and compare them to other carcinomas of the oral cavity.

## Material and Methods

 For the research process, we used different combinations of the keywords: "gingiva", "gum", "oral cancer", "oral squamous cell carcinoma" eta "oral carcinoma". Only registers of the last five years were included. Experimental studies and those without specific features on squamous cell carcinoma of the gingiva were not selected. Finally, 15 articles matched inclusion criteria.

## Results

 More than half of the cases of gingival carcinoma were diagnosed at advanced stages (60.04%), as were the buccal mucosa and palate ones; unlike the tongue, lip and palate (initial stages). Most carcinomas diagnosed in the gingiva were found at T4 (34.97%), similar to the tongue and buccal mucosa. On the contrary, T1 was the most frequent on the floor of the mouth, lip and palate. Also, N0 happened to be the most common stage in all locations, with no neck lymph node metastasis (96-99%). Overall, the main histopathological differentiation was well differentiated, except for the gingiva, where moderate had a higher incidence (66.85%).

## Conclusions

 Further studies are needed to analyze the actual prognostic validity of the TNM staging system, such as those that take into account individual and medical background of patients.

# Abstract: CAR-T cell therapies in head and neck malignancies. A review and update

**Authors:** M. Margaix Muñoz, M.G. Sarrión Pérez, A. Rubert, C. Gavaldá Esteve, J. Bagán, R. Poveda Roda

**Affiliations:** *Department of Stomatology. Oral Medicine University of Valencia. Valencia. Spain. Email: maria.margaix@uv.es*

## Objectives

 to offer a state-of-the-art view regarding recent advances achieved with Chimeric Antigen Receptors T cells (CAR-T) therapy on head and neck tumors, especially on squamous cell carcinoma (HNSCC). Recent treatment modalities with other immune cells, such as Natural Killer cells (CAR-NK) and macrophages (CAR-M), are also addressed.

## Material and Methods

 A literature review was conducted through PubMed-Medline and Scopus. Head and neck malignancies, squamous cell carcinoma, CAR-T, and immunotherapy were keywords. A total of 28 articles were found related to head and neck malignant tumors and CAR treatments.

## Results

 CARs are engineered receptors that graft a defined specificity onto an immune effector cell, typically T cells but also NK cells and macrophages, to enhance the immune response against tumor cells. Most of the data are provided by preclinical studies based on different targets (MUC1, CD44v6, EGFR, HER2, CD70, mesothelin) and a few clinical trials whose results have reported anti-tumor efficacy.

## Conclusions

 CAR therapies for locally advanced HNSCC management are in the early stages but might constitute a promising therapeutic strategy. The main barriers to be solved are the tumor microenvironment, limited choices of targeting proteins, cell infiltration into tumors, and short survival time in vivo.

# Abstract: Differential molecular features of gingival squamous cell carcinoma: a literature review

**Authors:** X. Marichalar Mendia, F. Mazhar Chaudhary Khan, I. Lafuente-Ibáñez de Mendoza, J.M. Aguirre Urizar

**Affiliations:** *Department of Nursery I. University of Basque Country/EHU. E-mail: xabier.marichalar@ehu.eus*

## Objectives

 To update the immunohistochemical differential aspects of gingival squamous cell carcinoma and compare them to other intraoral carcinomas.

## Material and Methods

 Three databases were used for the research process (PubMed, Web of Science, Scopus), using the following keywords: ‘gingiva’, ‘oral’, ‘carcinoma’, ‘oral cancer’ and ‘squamous cell’. The date of publication had to be five years ago. In vitro/in vivo studies were excluded, as well as those with no separate data on gingival squamous cell carcinoma. Only 4 articles were selected that matched the inclusion criteria, out of which 2 were obtained by handsearching.

## Results

 A total of 979 squamous cell carcinomas were analyzed, 250 of the gingiva (25.54%), 295 of the tongue (30.13%), 89 of the palate (9.09%) and 76 of the buccal mucosa (7.76%). The biomarkers investigated were: oncogenic molecules (p16, p53), cell structure molecules (TUBB3), and extracellular molecules related to tumor invasion (MMP-27, RANKL, OPG). Compared to other oral locations, the expression of these biomarkers was not been specific for gingival squamous cell carcinoma.

## Conclusions

 Further research, especially in the field of genetics and epigenetics and cell signaling pathways, will be needed in the future to better understand the oncogenesis of gingival squamous cell carcinoma.

# Abstract: Prevalence of acute oral mucosal damage secondary to the use of systemic antineoplastics: A systematic review and meta-analysis

**Authors:** N. Matala Álvarez, M.E. Rodriguez Fuentes, M. Pérez-Sayáns García, P. Gandara Vila, A. Blanco Carrión, A. García García

**Affiliations:** *Master of Oral Medicine, Oral Surgery and Implantology, University of Santiago de Compostela. España. Email: nasser_ab98@hotmail.com*

## Objectives

 The aim of this study was to determine the prevalence of acute oral mucosal toxicities in non-irradiated patients treated with systemic antineoplastics agents. The secondary objective was to find out differences in its prevalence among the different types of systemic antineoplastics.

## Material and Methods

 A systematic review and meta-analysis was performed. Articles from 2010 to July 2022 were retrieved and included if patients were adults undergoing oral assessment after administration of commercially available systemic antineoplastics. Data was extracted and pooled proportions were estimated using random-effect model method (Der Simonian and Lair).

## Results

 Eighty-two articles were included in the study. The overall prevalence of acute oral mucosal damage across studies was 38.2% (95% CI: 33.1%-43.3%). The prevalence was 42.9% (95% CI: 32.8%-53%) in patients treated with chemotherapy alone, 38% (95% CI: 29.1%-47%) in patients treated with a combination of chemotherapy and targeted therapies, and 32.1% (95% CI: 26.8%-37.5%) in targeted therapies alone-treated patients. No statistically significant differences were found in the prevalence of oral mucosal toxicities between the different types of systemic antineoplastic treatments.

## Conclusions

 Oral mucosal toxicity is a major side effect in non-irradiated cancer patients undergoing systemic antineoplastics.

# Abstract: Influence of prosthetic rehabilitation on salivary flow, ph and sense of comfort in patients treated for head and neck cancer

**Authors:** C. Moreno Soriano, C.S. Montoya Muñante, E. Jané Salas, J. López-López

**Affiliations:** *Department of Odontostomatology, UFR of Dentistry, Faculty of Medicine and Health Sciences (Dentistry), Bellvitge Campus, University of Barcelona. Email: carlosmorenosoriano@hotmail.com*

## Objectives

 To determine whether prosthetic rehabilitation with fixed and/or removable prostheses modifies salivary flow, pH and subjective sense of comfort in patients treated for head and neck cancer.

## Material and Methods

 Pilot clinical study that included patients treated for head and neck cancer candidates for prosthetic. They were divided into two groups, according to whether they received radiotherapy or not. The study consists of three sessions: T0 (before the prosthesis), T1 (one month after prosthesis was installed) and T6 (six months after prosthesis was installed). Sialometry (stimulated and unstimulated), salivary pH and sense of comfort were measured in every session.

## Results

 Twenty-one patients were studied. In the total sample, a significant increase was observed in both unstimulated (T0-T6) and stimulated (T0-T1, T0-T6, T1-T6). Although improvements were observed in both sialometries, no significant intra-group or inter-group differences were found. The pH increased between T0 and T6 in non-irradiated patients, and the change was significant with respect to irradiated patients. There was an improvement in the sense of comfort in both groups, without statistically significant differences.

## Conclusions

 More studies are needed, with a larger sample and more intra-group homogeneity, to be able to compare results and quantify the influence of prosthetic rehabilitation on salivary flow, pH and sense of comfort.

# Abstract: Correlation Between Salivary Metabolites, Clinical and Tobacco Consumption Data in Hookah Smokers

**Authors:** M.G.O. Alves, L.S. Theodoro, D. Aciole Barbosa, E. Greenfield, D.R. Cavalcanti, J. Dias Almeida, M.A. Mendes, F.B. Menegidio

**Affiliations:** *Universidade Estadual Paulista (UNESP), Instituto de Ciência e Tecnologia, Câmpus de São José dos Campos, São Paulo, Brazil. Email: monica.alves@unesp.br*

## Objectives

 The study aimed to evaluate the salivary metabolomic profile of hookah smokers, correlating it with clinical and tobacco consumption data. 

## Material and Methods

 The study groups consisted of 33 regular and exclusive hookah smokers, and 30 nonsmokers. Unstimulated whole saliva was collected and analyzed for salivary metabolites using Gas Chromatography-Tandem Mass Spectrometry (GC-MS/MS). Correlations were made with clinical data, tobacco consumption and cotinine levels measured by an immunoenzymatic test and with using statistical analysis on the MetaboAnalyst 6.0 platform.

## Results

 Partial least squares-discriminant analysis demonstrated a significant separation between the hookah group and the control group. Volcano plot analysis identified 32 statistically significant metabolites (p ≤0.05) correlated with clinical data and tobacco consumption in hookah group. Notably, lactic acid, fucose, sarcosine, and proline stood out due to their properties as oncological biomarkers and oncometabolites.

## Conclusions

 The correlation between salivary metabolites, clinical and tobacco consumption data revealed the expression of four important oncometabolites in the saliva of hookah users. *Acknowledgments: São Paulo State Research Foundation (FAPESP) for supporting Grant #2018/23248-1, Financing 03/2024 PROPe - UNESP and the Coordination of Higher-Level Staff Improvement (CAPES) for the master's scholarship.

# Abstract: Histopathological characteristics and presence of melanin in sporadic odontogenic keratocysts and associated with nevoid basal cell syndrome

**Authors:** A. Ortega Pinto, P. Reyes Ríos, I. Espinoza Santander

**Affiliations:** *Departamento de Patología y Medicina Oral, Facultad de Odontología, Universidad de Chile. Email: aortega@odontologia.uchile.cl*

## Objectives

 to compare histopathological characteristics and presence of melanin in odontogenic keratocysts associated with nevoid basal cellular syndrome (OKsy) and sporadic keratocysts (OKsp) in a sample of Chilean patients.

## Material and Methods

 Biopsies with a diagnosis of OKsy and OKsp from the Faculty of Dentistry of the University of Chile were analyzed. In Hematoxylin-Eosin stained slides, the presence of satellite cysts, nests and epithelial buds was evaluated. With immunohistochemistry, the presence and distribution of melanocytes was evaluated with Melan A antibody.

## Results

 A total of 29 OK were included in this study, 20 OKsp and 9 OKsy. The OKsy cases presented 88.8% of satellite cysts, epithelial nests or buds, while the OKsp cases only presented 40%. Melan A-positive cells occurred exclusively in OKsy in four of the nine cases. In all four cases the melanocytes were located in the basal stratum of the epithelium, with a dendritic appearance and separated by 5 to 11 basal cells.

## Conclusions

 The OKsy presented a higher percentage of satellite cysts, nests or epithelial buds and only in these cases melanocytes were observed in the cystic epithelium.

# Abstract: Influence of surgical margins in recurrence and survival rate in patients with oral squamous cell carcinoma: a systematic review and meta-analysis

**Authors:** A. Rubert, L. Bagan, Y. Jiménez, C. Gavaldá, J. Bagan, A. Proaño-Haro

**Affiliations:** *Department of Stomatology, Oral Medicine. European University of Valencia, Valencia, Spain. Email: andrea_rubert_ara@hotmail.com*

## Objectives

 Evaluate the impact of surgical margins on survival rate and local recurrence rate in patients with oral squamous cell carcinoma through a meta-analysis.

## Material and Methods

 An electronic search of Medline-PubMed, Web of Science and Scopus databases was conducted published up to December 2022. Odds ratios were estimated with the 95% confidence interval and forest plots were generated through random or fixed effect meta-analysis depending on heterogeneity. Sensitivity and publication bias analyze were performed using funnel plots and the Egger test.

## Results

 The odds ratio recurrence was higher in positive margins OR 2,673. IC95%: 1,937 – 3,689%. p = 0,000). The risk ratio was 0,656 (IC95%: 0,565 – 0,761. p = 0,000), negative margins are a protective factor in OSCC survival. Patients with negative surgical margins have a 1.52 times greater probability of 5-year survival than patients with positive surgical margins.

## Conclusions

 Surgical margins are a prognostic factor of survival in patients with oral squamous cell carcinoma. Local recurrence rate is significantly lower and survival rate is significantly higher in patients with negative surgical margins (≥5mm) in comparison with positive surgical margins patients (<1mm).

# Abstract: Performance in Identifying Potentially Malignant Oral Disorders, Oral Cancer and Benign Oral Lesions Among Dental Students in Chile

**Authors:** G. Ojeda, J.C. Salinas, C. Morales, D. Adorno, A. Ortega, I. Espinoza

**Affiliations:** *Facultad de Odontología, Universidad de Chile. Chile. Email: juancsalinas@odontologia.uchile.cl*

## Objectives

 To analyze the ability of dental students to accurately identify oral mucosal lesions, particularly potentially malignant oral disorders (PMOD), oral cancer and benign lesions.

## Material and Methods

 112 dental students in their final year of dental training at the University of Chile were tasked with diagnosing 33 cases of oral mucosal lesions. These cases were obtained from the records of the Oral Medicine Clinic in the Dental Clinic of the same university. The evaluation was through digital images in a face-to-face meeting and comprised 4 cases of PMOD, 3 cases of oral cancer, and 26 oral benign lesions not identified. Diagnostic concordance with the gold standard (biopsy or specialist diagnosis) and accuracy were determined. Analyses were performed using STATA 16.0. Ethics committee approval was obtained.

## Results

 The students correctly diagnosed 36% of the total cases, with 56% accuracy in diagnosing PMOD, 32% in identifying oral cancer cases, and 35% in diagnosing benign oral lesions. Sensitivity of 37.4% and specificity of 29.6% for diagnosing oral cancer/potentially malignant disorders compared to benign oral mucosal lesions were calculated.

## Conclusions

 Dental students exhibit difficulties in identifying oral mucosal lesions, showing a higher proficiency in identifying PMOD compared to oral cancer and benign lesions.

# Abstract: Clinical study of 46 Head and Neck Lymphomas

**Authors:** M.G. Sarrión Pérez, F. Mañes Jiménez, Y. Jiménez, C. Gavaldá, L. Bagán, J. Bagán

**Affiliations:** *Department of Stomatology, Oral Medicine. University of Valencia. Valencia, Spain. Email: m.gracia.sarrion@uv.es*

## Objectives

 to analyze a series of clinical cases of lymphomas with primary manifestation in the head and neck region.

## Material and Methods

 A retrospective observational study was carried out in the University General Hospital of Valencia and in the Oral Medicine clinics of the University of Valencia. The medical records of patients with a diagnosis of head and neck lymphoma who attended these centers as their first visit were collected and the following data were recorded: age and sex, histological type of lymphoma, location, extracervical organ involvement, symptoms, and stage according to the Lugano classification. A descriptive analysis was performed with the SPSS program.

## Results and Conclusions

 A total of 46 patients were included. The 60.9% were women and the mean age of involvement was 57 years. Diffuse large B-cell lymphomas accounted for 43.6%, followed much less frequently by follicular lymphomas (15.2%). The most affected site was Waldeyer's ring (39.1%). Only 8.7% of patients had B symptoms at the time of diagnosis. All patients were diagnosed at localized stage I and II, i.e. with a single extralymphatic lesion or with lymph nodes on the same side of the diaphragm.

# Abstract: Conservative management of osteorradionecrosis. Systematic review

**Authors:** H. Sliman, F. Alegria Riquelme, C.J. Omaña Cepeda, C. Moreno Soriano, C.S. Montoya Muñante, E. Jané Salas

**Affiliations:** *Facultat de Medicina i Ciències de la Salut. UFR Odontología, Campus Universitari Bellvitge. Universitat de Barcelona- España. Email: hibasliman@icloud.com*

## Introduction

 ORN is a serious and common complication in which the irradiated bone becomes devitalized and exposed through the overlying skin or mucosa, persisting without healing for more than 3 months, in the absence of recurrent disease. High doses of radiation (>60 Gy) and mechanical and infectious trauma are the main triggering factors.

## Objectives

 To review the prognosis and treatment of osteoradionecrosis (ORN).

## Material and Methods

 We present two cases of men, aged 52 and 58 years, with a history of oral squamous cell carcinoma (OSCC) of the tongue treated with radiotherapy (RT), who subsequently developed ORN. For the review, PubMed was searched using the keywords "osteoradionecrosis" AND "management" for articles published in the last 10 years.

## Results

 Of a total of 346 articles from the initial search, 6 articles related to the topic were obtained.

## Conclusions

 The avoidance of irritants such as tobacco, alcohol, and the use of dental prostheses is prioritized, along with the regular application of Chlorhexidine 0.02%. Additionally, antibiotic treatment is currently complemented with Pentoxifylline 400 mg and Tocopherol 500 IU due to their proven antioxidant and antifibrotic properties.

# Abstract: Proliferative multifocal leukoplakia and its biomarkers: Systematic review

**Authors:** D. Sotorra Figuerola, I. Lafuente-Ibáñez de Mendoza, X. Marichalar Mendia, J.M. Aguirre Urizar

**Affiliations:** *Department of Stomatology. University of Basque Country/EHU. E-mail: sotorrad@gmail.com.*

## Objectives

 To review and analyze the main biomarkers for proliferative multifocal leukoplakia (PML).

## Material and Methods

 A bibliographical search of the PubMed, Embase, Web of Science and Scopus databases was conducted. The entry keyword terms were: ([proliferative AND verrucous] OR multifocal) AND [leukoplakia OR leucoplakia] AND [tumor markers OR biomarkers OR immunohistochemistry]. We only selected studies were at least one biomarker was investigated in relation to PML, including retrospective case-controls, case-series and clinical studies, published since 1985.

## Results

 In total, 96 records were retrieved but only 15 articles were selected for the review. The most common biomarkers were DNA ploidy, Ki67, p53 and human papillomavirus (HPV). DNA ploidy was the sole one to show a predictive value for a PML development. Other biomarkers like PD-L1 have also been proposed as therapeutic target.

## Conclusions

 In spite of being several studies on biomarkers for oral cancer and conventional leukoplakia, there are just a few regarding PML. Due to its high risk of malignant development, it is crucial to invest more resources to find a predictive biomarker for PML.

# Abstract: Identification and elimination of risk factors in the management of low-risk oral potentially malignant disorders (OPMD)

**Authors:** A. Troiano, A. Nobili, L. DiVittorio, C. Digregorio, C. Ferdeghini, M. Roghi

**Affiliations:** *1Department of Oral Pathology, Istituto Stomatologico Italiano, Milan, Italy. Email: alessiotroiano12@gmail.com*

## Introduction

 OPMDs are mucosal diseases (including leukoplakia, erythroplakia, OLP, CHC, etc.) characterized by an increased risk of OSCC development. The main risk factors are: tobacco, alcohol, infections, and long-term trauma. The management of OPMDs relies on risk modification, active surveillance, and targeted biopsies.

## Case Reports

 Three patients, who clinically presented potentially malignant lesions, were selected. They had definite risk factors: smoke, alcohol at meal, and traumatic incongruous rehabilitations. A punch biopsy examination was performed and histological examination showed mild dysplasia (low-risk lesions). The cases clinically presented as leukoplakia. The approach initially involved the elimination of traumatic factors, sometimes with the aid of mouthguards. Hence, an incisional biopsy was performed, and persistent lesions were radicalized. Concurrently, smoking and alcohol consumption were gradually eliminated, thus the healing of the lesions could be observed.

## Conclusions

 Given that management strategies should depend on characteristics of the lesion (low- vs-high-risk), an effective approach must be multifactorial, not only with interventional surgery, but also through the progressive and complete elimination of every risk factor.

# Abstract: Epidemiological trends in oral cancer: beyond the traditional risk factors

**Authors:** B.C.D. Martins, T.D. Deps, A.C.F. de Araujo, A.C. Gomes Henriques Leitão, D.R. Camisasca, L.A.P. de Barros

**Affiliations:** *Master's student in the Graduate Program in Dental Sciences – UFES. Email: barbaracamposmartins@gmail.com*

## Objectives

 Demonstrate epidemiological patterns subject to the development of oral cancer distinct from conventional patterns associated with pathogenesis - tobacco/alcohol.

## Material and Methods

 Bibliographic survey in databases with the search strategy: "oral cancer AND risk factors AND prevalence", from 2015 to 2023. 

## Results

 30 articles were selected in English. The most prevalent profiles are men over 50-years-old, smokers and alcoholics. However, there is a documented trend of growth in young women with possible comorbidities related to the fragility of the immune system; in patients with dental implants suffering from peri-implantitis and consequent metal leaching in the oral cavity; in the oropharyngeal region of young individuals caused by human papillomavirus facilitated by sexual behavior with multiple partners, and increasing use of electronic cigarettes in this group; patients with proliferative verrucous leukoplakia, an oral potentially malignant disorders, with high potential for malignant transformation; furthermore, reports indicate malignant transformation in patients with chronic mechanical irritations.

## Conclusions

 Besides conventional tobacco smoking associated with oral carcinogenesis, other profiles susceptible to oral cancer development must be outlined in order to broaden promotion, prevention, and early detection strategies of this disease, favoring the reduction of incidence rates and diagnoses at advanced stages.
